# Supplementary material for: Maternal Iodine Status and Birth Outcomes: A Systematic Literature Review and Meta-Analysis
Source: Nutrients. 2023 Jan 12;15(2):387. doi: 10.3390/nu15020387 (PMC9865661; doi:10.3390/nu15020387)

Supplementary material

Supplementary tables

Supplementary Table S1: Full search strategies
Supplementary Table S2: List of excluded full text articles, with reasons
Supplementary Table S3: Covariate adjustment and Newcastle-Ottawa quality assessment for included studies
Supplementary Table S4: Summary of subgroup analyses for UIC and birth weight
Supplementary Table S5: Summary of subgroup analyses for UIC and SGA
Supplementary Table S6: Summary of subgroup analyses for UIC and preterm delivery

Supplementary figures

Supplementary Figure S1a: Birth weight and iodide intake forest plot for linear trend
Supplementary Figure S1b: Birth weight and iodide intake nonlinear trend
Supplementary Figure S2a: Low birth weight and UIC forest plot for dichotomous comparison
Supplementary Figure S2b: Low birth weight and UIC forest plot for linear trend
Supplementary Figure S2c: Low birth weight and UIC nonlinear trend
Supplementary Figure S3a: Macrosomia and UIC forest plot for dichotomous comparison
Supplementary Figure S3b: Macrosomia and UIC forest plot for linear trend
Supplementary Figure S3c: Macrosomia and UIC nonlinear trend
Supplementary Figure S4: SGA and iodide intake forest plot for linear trend
Supplementary Figure S5a: Birth length and UIC forest plot for dichotomous comparison
Supplementary Figure S5b: Birth length and UIC forest plot for linear trend
Supplementary Figure S5c: Birth length and UIC nonlinear trend
Supplementary Figure S6a: Head circumference and UIC forest plot for dichotomous comparison
Supplementary Figure S6b: Head circumference and UIC forest plot for linear trend
Supplementary Figure S6c: Head circumference and UIC nonlinear trend
Supplementary Figure S7a: Head circumference and I:Cr forest plot for dichotomous comparison
Supplementary Figure S7b: Head circumference and I:Cr forest plot for linear trend
Supplementary Figure S7c: Head circumference and I:Cr nonlinear trend
Supplementary Figure S8: Preterm delivery and iodide intake forest plot for linear trend
Supplementary Figure S9: Spontaneous abortion and UIC forest plot for linear trend
Supplementary Figure S10: Association between UIC and birth weight by mean gestation when urine sample provided
Supplementary Figure S11: Association between UIC and birth weight by median UIC of study population
Supplementary Figure S12: Association between UIC and birth weight by income status of country
Supplementary Figure S13: Association between UIC and birth weight by adjustment for potential confounding
Supplementary Figure S14: Association between UIC and birth weight by Newcastle-Ottawa quality assessment score for selection
Supplementary Figure S15: Association between UIC and birth weight by Newcastle-Ottawa quality assessment score for comparability
Supplementary Figure S16: Association between UIC and birth weight by Newcastle-Ottawa quality assessment score for outcome
Supplementary Figure S17: Association between UIC and SGA by mean gestation when urine sample provided
Supplementary Figure S18: Association between UIC and SGA by median UIC of study population
Supplementary Figure S19: Association between UIC and SGA by income status of country

Supplementary Figure S20: Association between UIC and SGA by adjustment for potential confounding
Supplementary Figure S21: Association between UIC and SGA by Newcastle-Ottawa quality assessment score for selection
Supplementary Figure S22: Association between UIC and SGA by Newcastle-Ottawa quality assessment score for comparability
Supplementary Figure S23: Association between UIC and SGA by Newcastle-Ottawa quality assessment score for outcome
Supplementary Figure S24: Association between UIC and preterm delivery by mean gestation when urine sample provided
Supplementary Figure S25: Association between UIC and preterm delivery by median UIC of study population
Supplementary Figure S26: Association between UIC and preterm delivery by income status of country
Supplementary Figure S27: Association between UIC and preterm delivery by adjustment for potential confounding
Supplementary Figure S28: Association between UIC and preterm delivery by Newcastle-Ottawa quality assessment score for selection
Supplementary Figure S29: Association between UIC and preterm delivery by Newcastle-Ottawa quality assessment score for comparability
Supplementary Figure S30: Association between UIC and preterm delivery by Newcastle-Ottawa quality assessment score for outcome
Supplementary Figure S31: Contour-enhanced funnel plots for UIC and birth weight
Supplementary Figure S32: Contour-enhanced funnel plots for UIC and preterm delivery

Supplementary Table S1: Full search strategies.

MEDLINE

1. exp cohort studies/
2. cohort$.tw.
3. controlled clinical trial.pt.
4. epidemiologic methods/
5. limit 4 to yr=1971-1988
6. or/1-3,5
7. (animals not (humans and animals)).sh.
8. 6 not 7

9. pregnan$.ti,ab.
10. matern$.ti,ab.
11. birth.ti,ab.
12. neonat$.ti,ab.
13. or/9-12

14. exp iodine/
15. iodine.ti,ab.
16. iodide?.ti,ab.
17. iodate?.ti,ab.
18. UIC.ti,ab.
19. UIE.ti,ab.
20. iodis$.ti,ab.
21. iodiz$.ti,ab.
22. or/14-21

23. exp miscarriage/
24. miscarriage.ti,ab.
25. exp spontaneous abortion/
26. spontaneous abortion.ti,ab.
27. exp preterm birth/
28. ((preterm or pre-term) adj2 (birth or delivery)).ti,ab.
29. ((f?etal or f?etus or intrauterine) adj2 (growth or restriction or retardation)).ti,ab.
30. FGR.ti,ab.
31. IUGR.ti,ab.
32. (small adj2 gestation$).ti,ab.
33. SGA.ti,ab.
34. exp birth weight/
35. (birth adj2 weight$).ti,ab.
36. birthweight.ti,ab.
37. (head adj3 circumference).ti,ab.
38. or/23-37

39. 8 and 13 and 22 and 38

EMBASE

1. exp cohort analysis/
2. exp longitudinal study/
3. exp prospective study/
4. exp follow up/
5. cohort$.tw.
6. or/1-5
7. (animals not (humans and animals)).sh.
8. 6 not 7

9. pregnan$.ti,ab.
10. matern$.ti,ab.
11. birth.ti,ab.
12. neonat$.ti,ab.
13. or/9-12

14. exp iodine/
15. iodine.ti,ab.
16. iodide?.ti,ab.
17. iodate?.ti,ab.
18. UIC.ti,ab.
19. UIE.ti,ab.
20. iodis$.ti,ab.
21. iodiz$.ti,ab.
22. or/14-21

23. exp miscarriage/
24. miscarriage.ti,ab.
25. exp spontaneous abortion/
26. spontaneous abortion.ti,ab.
27. exp preterm birth/
28. ((preterm or pre-term) adj2 (birth or delivery)).ti,ab.
29. ((f?etal or f?etus or intrauterine) adj2 (growth or restriction or retardation)).ti,ab.
30. FGR.ti,ab.
31. IUGR.ti,ab.
32. (small adj2 gestation$).ti,ab.
33. SGA.ti,ab.
34. exp birth weight/
35. (birth adj2 weight$).ti,ab.
36. birthweight.ti,ab.
37. (head adj3 circumference).ti,ab.
38. or/23-37

39. 8 and 13 and 22 and 38

Supplementary **Table S2: List of excluded full text articles, with reasons.**

Not primary research (n=1) [1]

Conference abstract (n=5) [2-6]

Not a cohort study (n=2) [7,8]

No relevant exposure (n=10) [9-18]

No relevant outcome (n=2) [19,20]

No comparison of exposure (n=4) [21-24]

No full text available (n=3) [25-27]

Data replicated in another publication (n=2) [28,29]

References

1. Emmett, P.M.; Jones, L.R.; Golding, J. Pregnancy diet and associated outcomes in the Avon Longitudinal Study of Parents and Children. *Nutrition Reviews* **2015**, *73*, 154-174, doi:https://dx.doi.org/10.1093/nutrit/nuv053.
2. Abel, M.H.; Caspersen, I.H.; Magnus, P.; Alexander, J.; Meltzer, H.M.; Brantsaeter, A.L. Mild-to-moderate iodine deficiency is associated with lower birthweight and increased risk of preterm delivery in the Norwegian Mother, Father, and Child Cohort Study. *Norsk Epidemiologi* **2019**, *28*, 85.
3. Abel, M.H.; Caspersen, I.H.; Sengpiel, V.; Jacobsson, B.; Magnus, P.M.; Alexander, J.; Meltzer, H.M.; Brantsaeter, A.L. Mild-to-moderate iodine deficiency is associated with lower birthweight and increased risk of preterm delivery in a large Norwegian pregnancy cohort. *Proceedings of the Nutrition Society* **2020**, *79*, doi:https://dx.doi.org/10.1017/S0029665120003572.
4. McKeating, D.; Bennett, W.; Clifton, V.; Zhang, P.; Perkins, A. Elemental metabolomics to identify pregnancy risk factors and predict gestational outcomes. *Placenta* **2019**, *83*, e97-e98, doi:https://dx.doi.org/10.1016/j.placenta.2019.06.308.
5. Shan, Z.; Li, C.; Teng, W. Effects of iodine nutrition of pregnant women on obsetric complication during pregnancy and intellectual development of offspring. *European Thyroid Journal* **2018**, *7*, 4, doi:https://dx.doi.org/10.1159/000491542.
6. Tang, Q.; Run, H.; Lu, Y. Iodine nutrition during pregnancy and related neonatal physical development in Shanghai. *Journal of Pediatric Gastroenterology and Nutrition* **2016**, *63*, S33, doi:https://dx.doi.org/10.1097/01.mpg.0000503536.79797.66.
7. Behrooz, H.G.; Tohidi, M.; Mehrabi, Y.; Behrooz, E.G.; Tehranidoost, M.; Azizi, F. Subclinical hypothyroidism in pregnancy: intellectual development of offspring. *Thyroid* **2011**, *21*, 1143-1147, doi:https://dx.doi.org/10.1089/thy.2011.0053.
8. Gargari, S.S.; Fateh, R.; Bakhshali-Bakhtiari, M.; Saleh, M.; Mirzamoradi, M.; Bakhtiyari, M. Maternal and neonatal outcomes and determinants of iodine deficiency in third trimester of pregnancy in an iodine sufficient area. *BMC Pregnancy and Childbirth* **2020**, *20*, 174, doi:https://dx.doi.org/10.1186/s12884-020-02863-6.
9. Abreu, S.; Santos, P.C.; Montenegro, N.; Mota, J. Relationship between dairy product intake during pregnancy and neonatal and maternal outcomes among Portuguese women. *Obesity Research and Clinical Practice* **2017**, *11*, 276-286, doi:https://dx.doi.org/10.1016/j.orcp.2016.07.001.

10. He, X.; Yan, Q.; Liu, C.; Wang, Z.; Liao, P.; Liu, T.; Shi, Z.; Song, Q.; Cui, X.; Wang, W., et al. Association of maternal thyroid dysfunction and autoimmunity with adverse birth outcomes. *Endocrine Connections* **2022**, *11*, e210599, doi:https://dx.doi.org/10.1530/EC-21-0599.
11. Knight, B.A.; Shields, B.M.; He, X.; Pearce, E.N.; Braverman, L.E.; Sturley, R.; Vaidya, B. Effect of perchlorate and thiocyanate exposure on thyroid function of pregnant women from South-West England: A cohort study. *Thyroid Research* **2018**, *11*, 9, doi:https://dx.doi.org/10.1186/s13044-018-0053-x.
12. McAlpine, J.M.; McKeating, D.R.; Vincze, L.; Vanderlelie, J.J.; Perkins, A.V. Essential Mineral Intake During Pregnancy and Its Association With Maternal Health and Birth Outcomes in South East Queensland, Australia. *Nutrition and Metabolic Insights* **2019**, *12*, 1178638819879444, doi:https://dx.doi.org/10.1177/1178638819879444.
13. Melero, V.; Runkle, I.; de la Torre, N.G.; De Miguel, P.; Valerio, J.; Del Valle, L.; Barabash, A.; Sanabria, C.; Moraga, I.; Familiar, C., et al. The consumption of food-based iodine in the immediate pre-pregnancy period in madrid is insufficient. San carlos and pregnancy cohort study. *Nutrients* **2021**, *13*, 4458, doi:https://dx.doi.org/10.3390/nu13124458.
14. Mills, J.L.; Mehnaz, A.; Louis, G.M.B.; Kannan, K.; Weck, J.; Wan, Y.; Maisog, J.; Giannakou, A.; Sundaram, R. Pregnancy loss and iodine status: The LIFE prospective cohort study. *Nutrients* **2019**, *11*, 534, doi:https://dx.doi.org/10.3390/nu11030534.
15. Murillo-Llorente, M.T.; Llorca-Colomer, F.; Perez-Bermejo, M. Relationship between thyroid status during the first trimester of pregnancy and neonatal well-being. *Nutrients* **2021**, *13*, 1-12, doi:https://dx.doi.org/10.3390/nu13030872.
16. Purdue-Smithe, A.C.; Mannisto, T.; Bell, G.A.; Mumford, S.L.; Liu, A.; Kannan, K.; Kim, U.J.; Suvanto, E.; Surcel, H.M.; Gissler, M., et al. The joint role of thyroid function and iodine status on risk of preterm birth and small for gestational age: A population-based nested case-control study of Finnish women. *Nutrients* **2019**, *11*, 2573, doi:https://dx.doi.org/10.3390/nu11112573.
17. Wang, Z.; Zhao, S.; Cui, X.; Song, Q.; Shi, Z.; Su, J.; Zang, J. Effects of dietary patterns during pregnancy on preterm birth: A birth cohort study in Shanghai. *Nutrients* **2021**, *13*, 2367, doi:https://dx.doi.org/10.3390/nu13072367.
18. Horan, M.K.; McGowan, C.A.; Gibney, E.R.; Donnelly, J.M.; McAuliffe, F.M. The association between maternal dietary micronutrient intake and neonatal anthropometry - Secondary analysis from the ROLO study. *Nutrition Journal* **2015**, *14 (1) (no pagination)*.
19. Aguayo, A.; Grau, G.; Vela, A.; Aniel-Quiroga, A.; Espada, M.; Martul, P.; Castano, L.; Rica, I. Urinary iodine and thyroid function in a population of healthy pregnant women in the North of Spain. *Journal of Trace Elements in Medicine and Biology* **2013**, *27*, 302-306, doi:https://dx.doi.org/10.1016/j.jtemb.2013.07.002.
20. Diemert, A.; Lezius, S.; Pagenkemper, M.; Hansen, G.; Drozdowska, A.; Hecher, K.; Arck, P.; Zyriax, B.C. Maternal nutrition, inadequate gestational weight gain and birth weight: Results from a prospective birth cohort. *BMC Pregnancy and Childbirth* **2016**, *16*, 224, doi:https://dx.doi.org/10.1186/s12884-016-1012-y.
21. Korevaar, T.I.M.; Schalekamp-Timmermans, S.; De Rijke, Y.B.; Visser, W.E.; Visser, W.; De Muinck Keizer-Schrama, S.M.P.F.; Hofman, A.; Ross, H.A.; Hooijkaas, H.; Tiemeier, H., et al. Hypothyroxinemia and TPO-antibody positivity are risk factors for premature delivery: The generation R study. *Journal of Clinical Endocrinology and Metabolism* **2013**, *98*, 4382-4390, doi:https://dx.doi.org/10.1210/jc.2013-2855.
22. Liu, H.; Shan, Z.; Li, C.; Mao, J.; Xie, X.; Wang, W.; Fan, C.; Wang, H.; Zhang, H.; Han, C., et al. Maternal subclinical hypothyroidism, thyroid autoimmunity, and the risk of miscarriage: A prospective cohort study. *Thyroid* **2014**, *24*, 1-8, doi:https://dx.doi.org/10.1089/thy.2014.0029.
23. Xu, F.; Xu, S.; Shao, X.; Wu, M.; Xu, Y.; Wang, L. Deficiency of calcium and microelements predict the risk of fetal growth restriction. *International Journal of Clinical and Experimental Medicine* **2017**, *10*, 7491-7499.

24. Menon, K.C.; Skeaff, S.A.; Thomson, C.D.; Gray, A.R.; Ferguson, E.L.; Zodpey, S.; Saraf, A.; Das, P.K.; Pandav, C.S. The effect of maternal iodine status on infant outcomes in an iodine-deficient Indian population. *Thyroid* **2011**, *21*, 1373-1380, doi:10.1089/thy.2011.0130.
25. Ardawi, M.S.M.; Nasrat, H.A.; Mustafa, B.E. Urinary iodine excretion and maternal thyroid function. During pregnancy and postpartum. *Saudi Medical Journal* **2002**, *23*, 413-422.
26. Sun, C.; Ruan, H.; Lu, Y.; Tang, Q. Maternal iodine nutrition during late pregnancy and neonatal physical development. *Chinese Journal of Clinical Nutrition* **2020**, *28*, 12-17, doi:https://dx.doi.org/10.3760/cma.j.cn115822-20190909-00134.
27. Olivares, J.L.; Olivi, G.I.; Verdasco, C.; Ortiz, V.A.; Mayer, M.A.; Cresto, J.C. Low iodine intake during pregnancy: relationship to placental development and head circumference in newborn. *Endocrinol Nutr* **2012**, *59*, 326-330, doi:10.1016/j.endonu.2011.12.005.
28. Alvarez-Pedrerol, M.; Guxens, M.; Mendez, M.; Canet, Y.; Martorell, R.; Espada, M.; Plana, E.; Rebagliato, M.; Sunyer, J. Iodine levels and thyroid hormones in healthy pregnant women and birth weight of their offspring. *European Journal of Endocrinology* **2009**, *160*, 423-429, doi:EJE-08-0716 [pii];10.1530/EJE-08-0716 [doi].
29. Hynes, K.L.; Otahal, P.; Hay, I.; Burgess, J.R. Mild iodine deficiency during pregnancy is associated with reduced educational outcomes in the offspring: 9-year follow-up of the gestational iodine cohort. *Journal of Clinical Endocrinology & Metabolism* **2013**, *98*, 1954-1962, doi:jc.2012-4249 [pii];10.1210/jc.2012-4249 [doi].

Supplementary Table S3: Covariate adjustment and Newcastle-Ottawa quality assessment for included studies

Author, year	Covariate adjustments	Selection	Comparability	Outcome
Abel, 2020	age, BMI, parity, smoking, gestation, sex, SES/education	****	**	**
Bienertová-Vašků, 2018		***	-	**
Charoenratana, 2015	age, BMI, parity, gestation, sex, SES/education, previous history	***	*	***
Chen, 2018	age, BMI, parity, smoking, gestation, race, SES/education, alcohol	****	**	**
Cui, 2022	age, SES/education, previous history	***	*	**
Dillon, 2000		***	-	*
Dong, 2021	gestation	***	-	**
Farebrother, 2020	age, BMI, parity, smoking, gestation, sex, race, SES/education	***	*	**
Ghassabian, 2014		****	-	**
Hynes, 2017		****	-	**
Kianpour, 2019		****	-	***
Lean, 2013		****	-	**
Leon, 2015	gestation, sex	****	**	**
Nazarpour, 2020	age, BMI	****	*	**
Ovadia, 2022	gestation, sex	***	-	**
Rydbeck, 2014		***	-	***
Snart, 2019	age, BMI, parity, gestation, sex, race, SES/education	****	**	**
Snart, 2020	BMI, parity, smoking, gestation, sex, race, SES/education, alcohol	****	**	**
Threapleton, 2021	BMI, parity, smoking, gestation, sex, race, SES/education, alcohol	****	**	**
Torlinska, 2018	age, BMI, parity, smoking, gestation, sex, race	****	**	**
Xiao, 2018	age, BMI, smoking	****	*	***
Yang, 2018	age, gestation, sex, SES/education	****	*	**
Yoganathan, 2015		***	-	***
Zhang, 2022	age, BMI, parity, gestation, sex, previous history	****	*	***

BMI = body mass index, SES = socio-economic status

Supplementary Table S4: Summary of subgroup analyses for UIC and birth weight

Subgroup	Meta-analysis of dichotomous UIC ≥150μg/L vs <150 μg/L			Meta-analysis of linear dose-response trend per 50μg/L UIC		
	n	Δ birth weight (g) (95% CI)	p	n	Δ birth weight (g) (95% CI)	p
Mean gestation when urine sample provided						
<18 weeks	7	-5 (-28, 19)		7	0 (-8, 9)	
18+ weeks	9	35 (-42, 112)	P=0.3	7	2 (-9, 13)	P=0.8
Median UIC of study population						
<150μg/L	8	9 (-15, 33)		7	3 (-5, 11)	
150+μg/L	5	69 (-52, 190)	P=0.3	5	6 (-4, 17)	P=0.6
Income status of country						
High income	6	21 (1, 42)		5	6 (-4, 17)	
Low or middle income	7	48 (-46, 142)	P=0.6	7	2 (-7, 10)	P=0.5
Adjustment for potential confounding						
No adjustment	2	100 (-141, 341)		2	11 (-2, 24)	
Any adjustment	11	5 (-22, 33)	P=0.4	10	2 (-5, 10)	P=0.3
Newcastle-Ottawa score: selection						
***	3	205 (169, 241)		3	7 (-2, 15)	
****	10	-2 (-26, 22)	P<0.001	9	2 (-6, 11)	P=0.5
Newcastle-Ottawa score: comparability						
-	2	100 (-141, 341)		2	11 (-2, 24)	
*	6	6 (-62, 73)		5	-4 (-11, 4)	
**	5	30 (-22, 83)	P=0.7	5	6 (-4, 16)	P=0.1

Newcastle-Ottawa score: outcome						
**	10	24 (-35, 83)		9	4 (-4, 11)	
***	3	57 (-92, 206)	P=0.7	3	6 (-1, 13)	P=0.7

Supplementary Table S5: Summary of subgroup analyses for UIC and SGA

	Meta-analysis of dichotomous UIC ≥150µg/L vs <150 µg/L			Meta-analysis of linear dose-response trend per 50µg/L UIC		
Subgroup	n	RR (95% CI)	p	n	RR (95% CI)	p
Mean gestation when urine sample provided						
<18 weeks	4	0.93 (0.74, 1.18)		4	1.00 (0.93, 1.07)	
18+ weeks	6	0.86 (0.75, 0.98)	P=0.6	6	0.96 (0.90, 1.02)	P=0.4
Median UIC of study population						
<150µg/L	5	0.89 (0.77, 1.02)		5	0.98 (0.94, 1.03)	
150+µg/L	3	0.77 (0.62, 0.95)	P=0.3	3	0.91 (0.81, 1.02)	P=0.2
Income status of country						
High income	5	0.89 (0.77, 1.04)		5	0.98 (0.94, 1.03)	
Low or middle income	3	0.77 (0.62, 0.95)	P=0.3	3	0.91 (0.81, 1.02)	P=0.2
Adjustment for potential confounding						
No adjustment		-			-	
Any adjustment	8	0.85 (0.75, 0.96)		8	0.96 (0.92, 1.01)	
Newcastle-Ottawa score: selection						
***	1	0.68 (0.39, 1.19)		1	0.82 (0.69, 0.97)	
****	7	0.86 (0.76, 0.97)	P=0.4	7	0.98 (0.94, 1.02)	P=0.05
Newcastle-Ottawa score: comparability						
*	3	0.77 (0.62, 0.95)		3	0.91 (0.81, 1.02)	
**	5	0.89 (0.77, 1.02)	P=0.3	5	0.98 (0.94, 1.03)	P=0.2
Newcastle-Ottawa score: outcome						
**	6	0.86 (0.76. 0.97)		6	0.98 (0.94, 1.02)	

***	2	0.72 (0.42, 1.23)	P=0.5	2	0.84 (0.72, 0.99)	P=0.08

Supplementary Table S6: Summary of subgroup analyses for UIC and preterm delivery

	Meta-analysis of dichotomous UIC ≥150µg/L vs <150 µg/L			Meta-analysis of linear dose-response trend per 50µg/L UIC		
Subgroup	n	RR (95% CI)	p	n	RR (95% CI)	p
Mean gestation when urine sample provided						
<18 weeks	7	0.84 (0.67. 1.06)		6	0.95 (0.89, 1.01)	
18+ weeks	8	0.91 (0.66, 1.26)	P=0.7	8	0.98 (0.91, 1.06)	P=0.5
Median UIC of study population						
<150µg/L	8	1.00 (0.81, 1.24)		8	0.99 (0.93, 1.06)	
150+µg/L	4	0.70 (0.53, 0.94)	P=0.06	4	0.92 (0.86, 1.00)	P=0.1
Income status of country						
High income	5	1.18 (0.94, 1.46)		5	1.04 (0.97, 1.12)	
Low or middle income	7	0.76 (0.59, 0.97)	P=0.01	7	0.94 (0.89, 0.99)	P=0.02
Adjustment for potential confounding						
No adjustment	2	1.29 (0.65, 2.58)		2	1.06 (0.93, 1.21)	
Any adjustment	10	0.85 (0.68, 1.06)	P=0.3	10	0.96 (0.90, 1.02)	P=0.2
Newcastle-Ottawa score: selection						
***	3	0.71 (0.36, 1.14)		3	0.94 (0.83, 1.06)	
****	9	0.92 (0.74, 1.15)	P=0.5	9	0.98 (0.92, 1.04)	P=0.5
Newcastle-Ottawa score: comparability						
-	2	1.29 (0.65, 5.58)		2	1.06 (0.93, 1.21)	
*	6	0.75 (0.57, 0.98)		6	0.93 (0.88, 0.99)	
**	4	1.15 (0.92, 1.45)	P=0.04	4	1.03 (0.94, 1.12)	P=0.08
Newcastle-Ottawa score: outcome						

**	8	0.96 (0.77, 1.19)		8	0.98 (0.92, 1.05)	
***	4	0.70 (0.45, 1.09)	P=0.2	4	0.94 (0.85, 1.04)	P=0.4

Supplementary Figure S1a: Birth weight and iodide intake forest plot for linear trend.

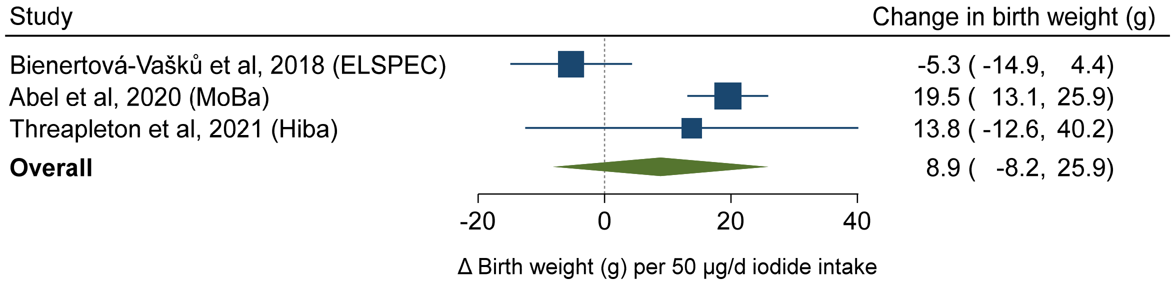

Supplementary Figure S1b: Birth weight and iodide intake nonlinear trend.

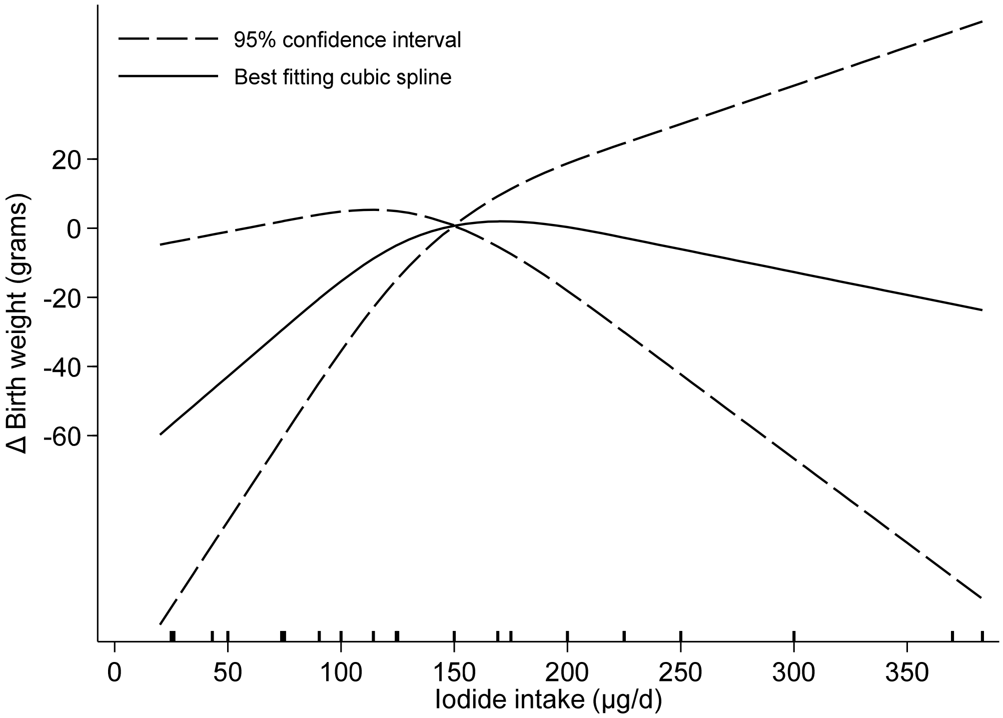

Supplementary Figure S2a: Low birth weight and UIC forest plot for dichotomous comparison.

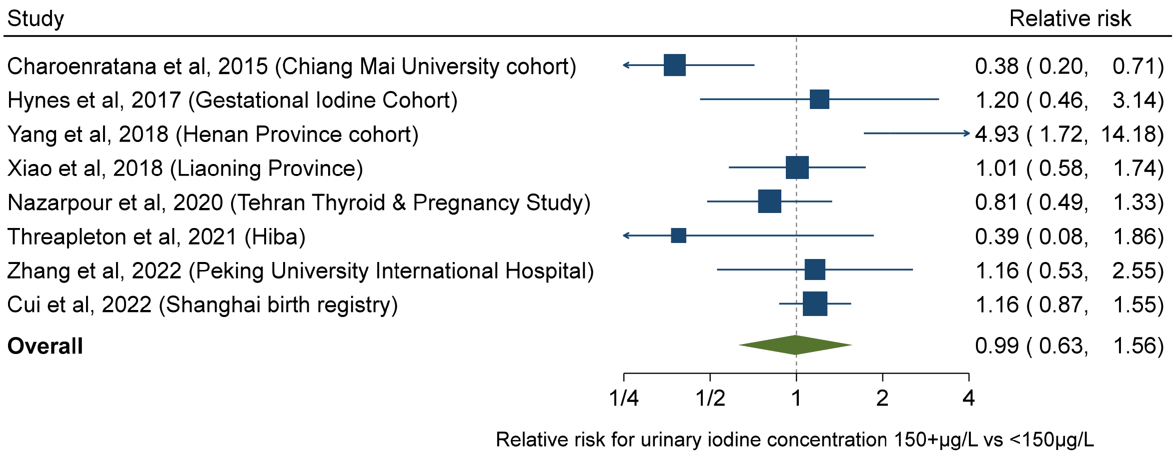

Supplementary Figure S2b: Low birth weight and UIC forest plot for linear trend.

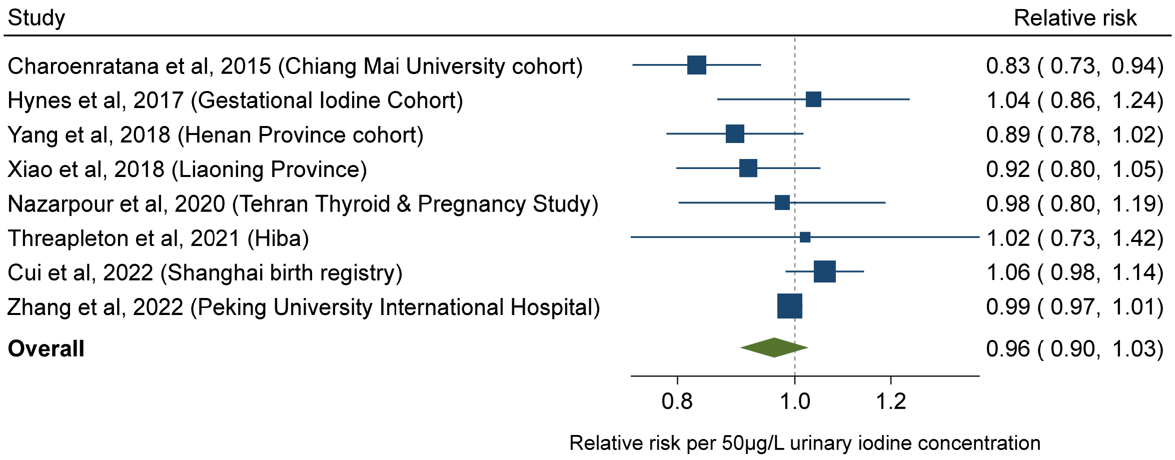

Supplementary Figure S2c: Low birth weight and UIC nonlinear trend.

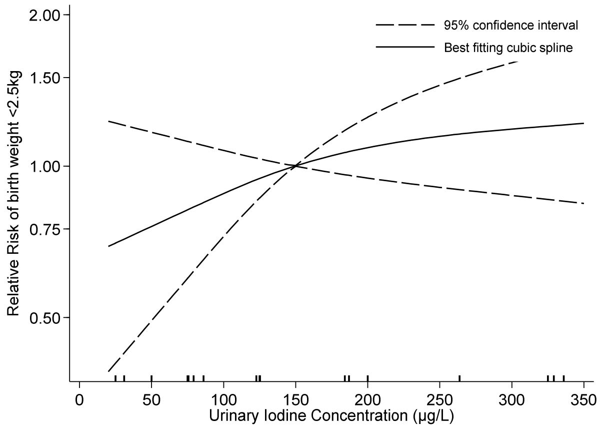

Supplementary Figure S3a: Macrosomia and UIC forest plot for dichotomous comparison.

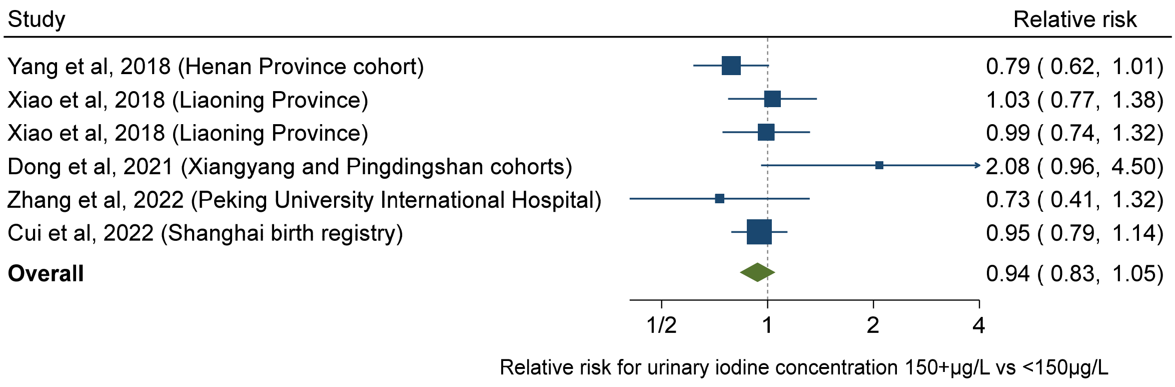

Supplementary Figure S3b: Macrosomia and UIC forest plot for linear trend.

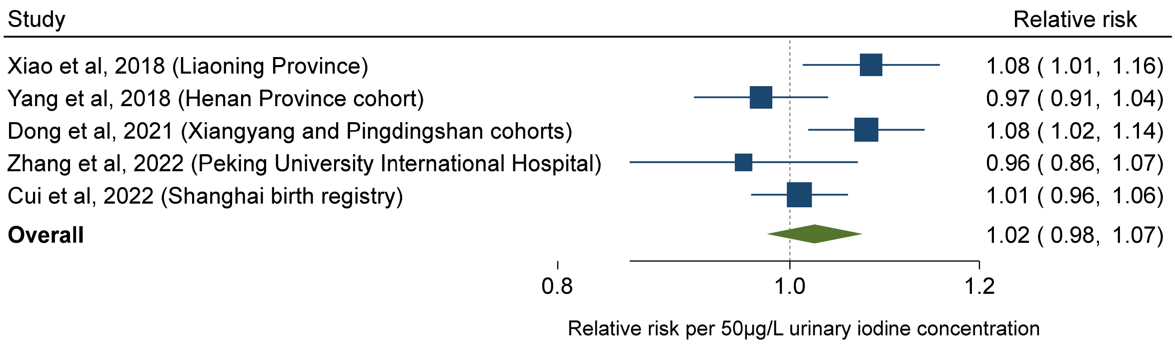

Supplementary Figure S3c: Macrosomia and UIC nonlinear trend.

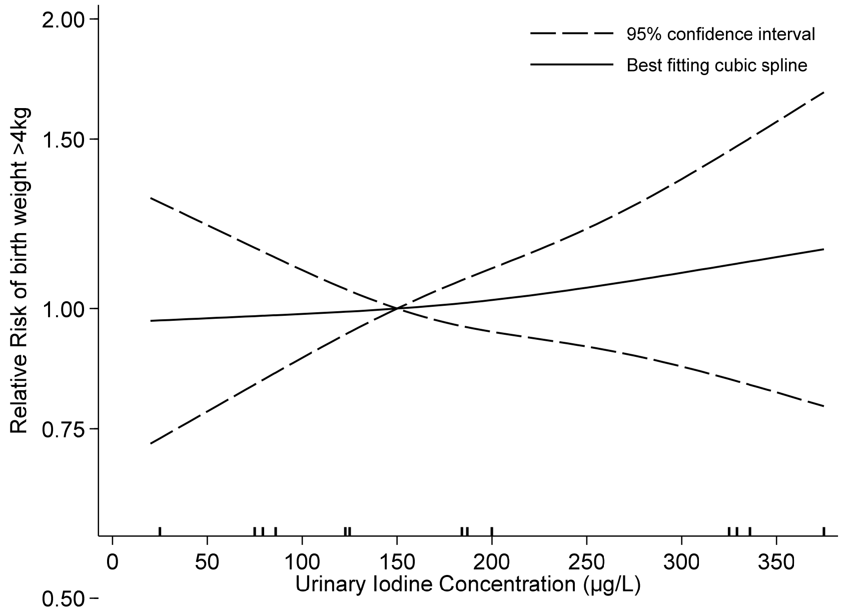

Supplementary Figure S4: SGA and iodide intake forest plot for linear trend

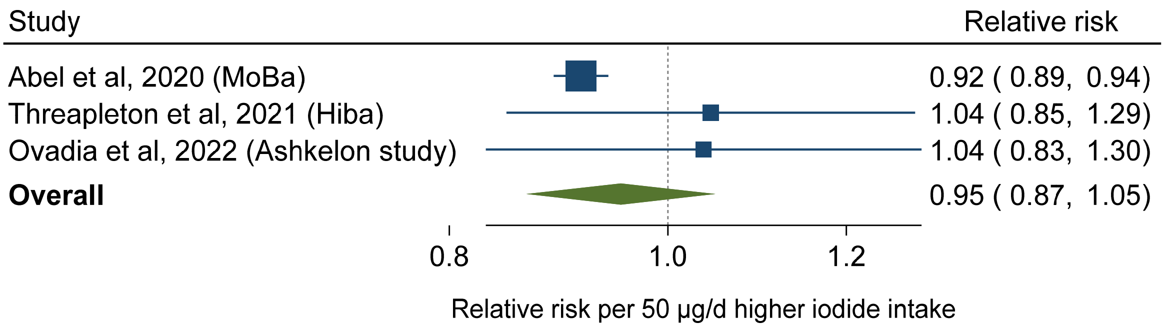

Supplementary Figure S5a: Birth length and UIC forest plot for dichotomous comparison.

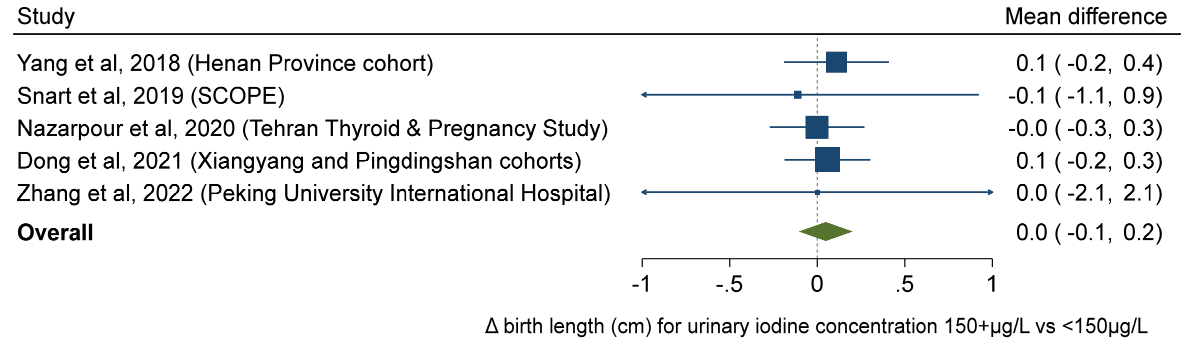

Supplementary Figure S5b: Birth length and UIC forest plot for linear trend.

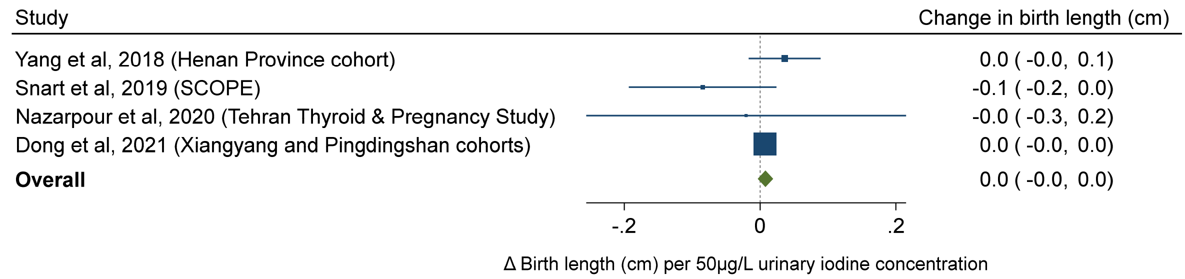

Supplementary Figure S5c: Birth length and UIC nonlinear trend.

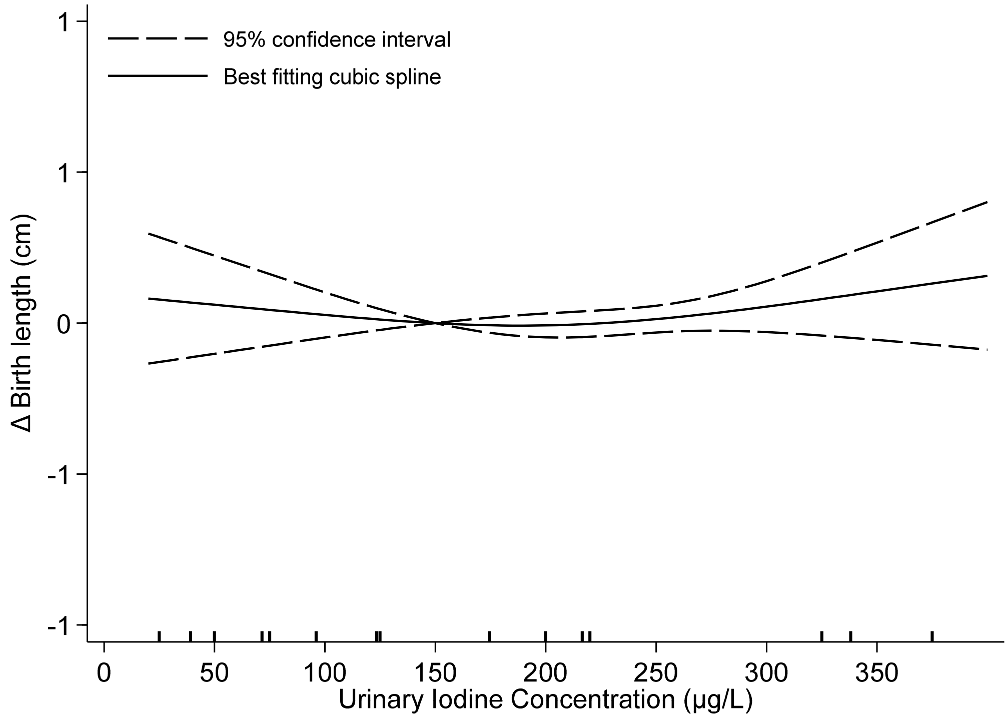

Supplementary Figure S6a: Head circumference and UIC forest plot for dichotomous comparison.

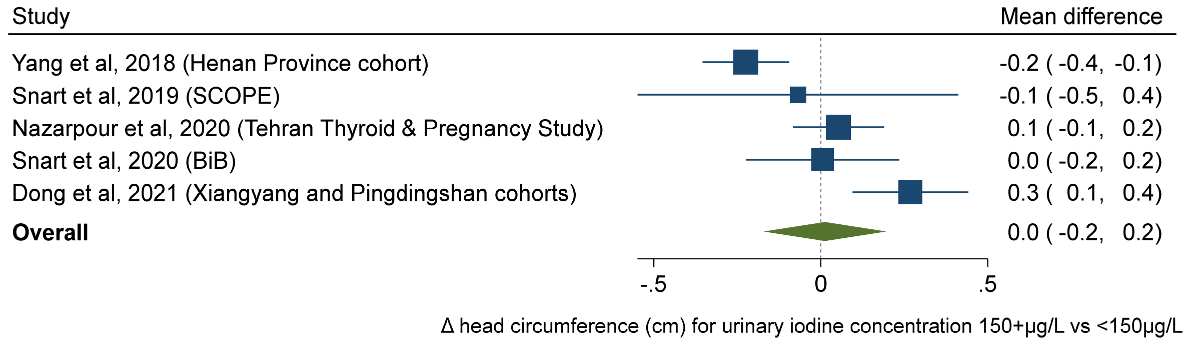

Supplementary Figure S6b: Head circumference and UIC forest plot for linear trend.

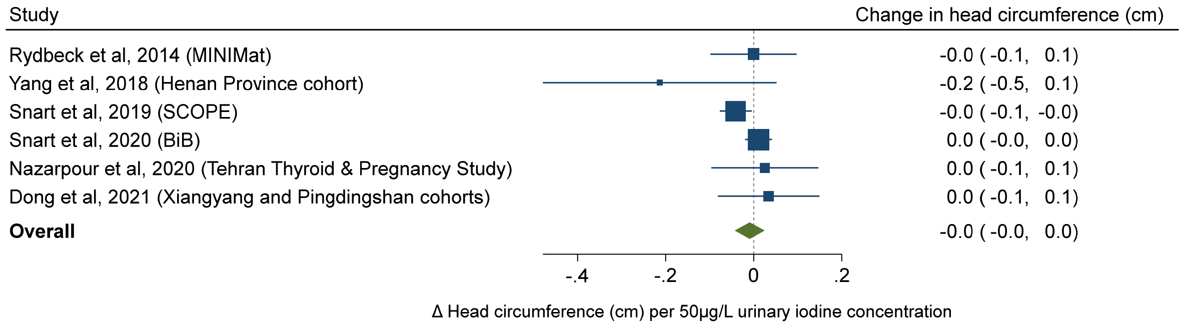

Supplementary Figure S6c: Head circumference and UIC nonlinear trend.

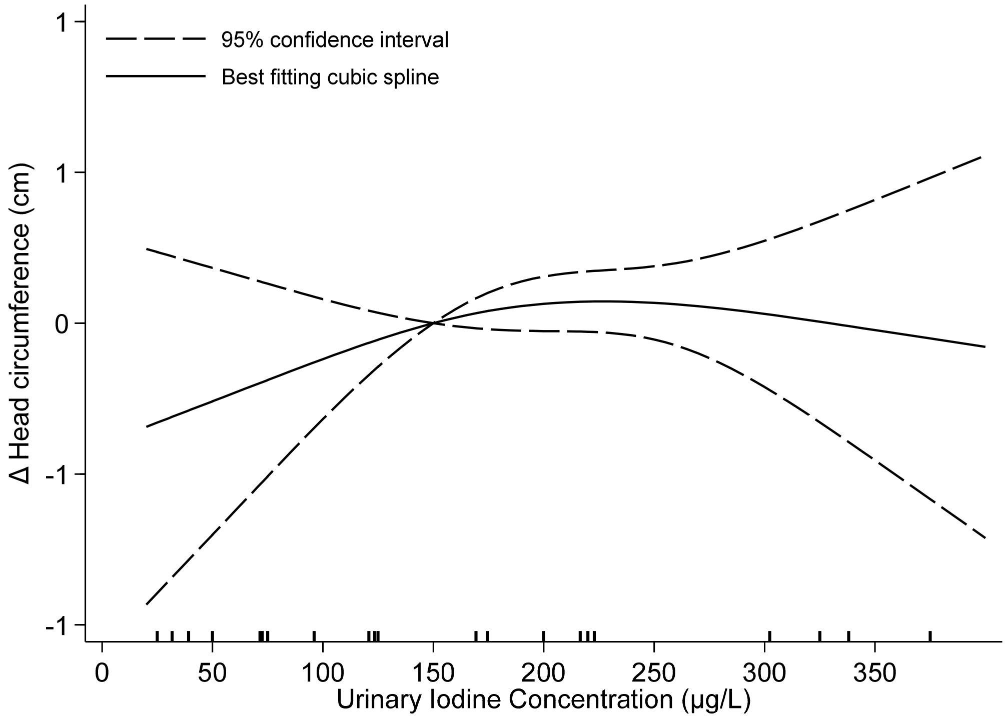

Supplementary Figure S7a: Head circumference and I:Cr forest plot for dichotomous comparison

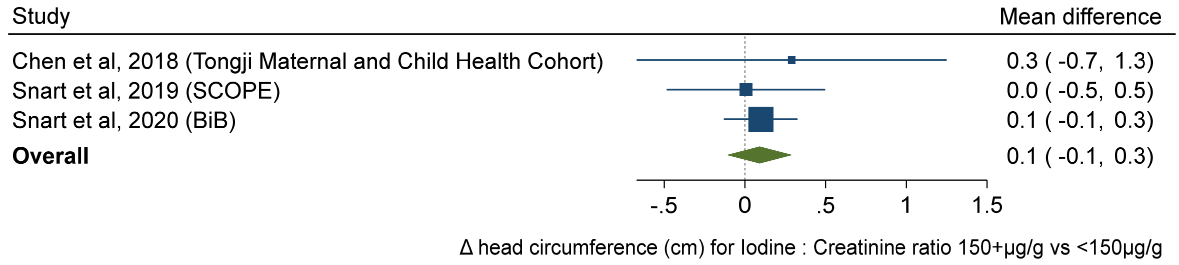

Supplementary Figure S7b: Head circumference and I:Cr forest plot for linear trend

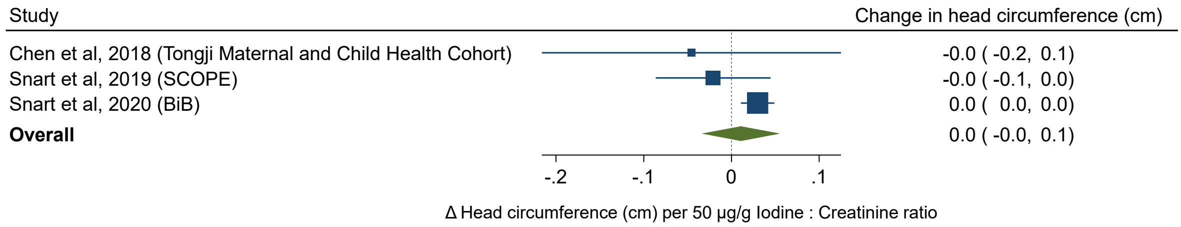

Supplementary Figure S7c: Head circumference and I:Cr nonlinear trend

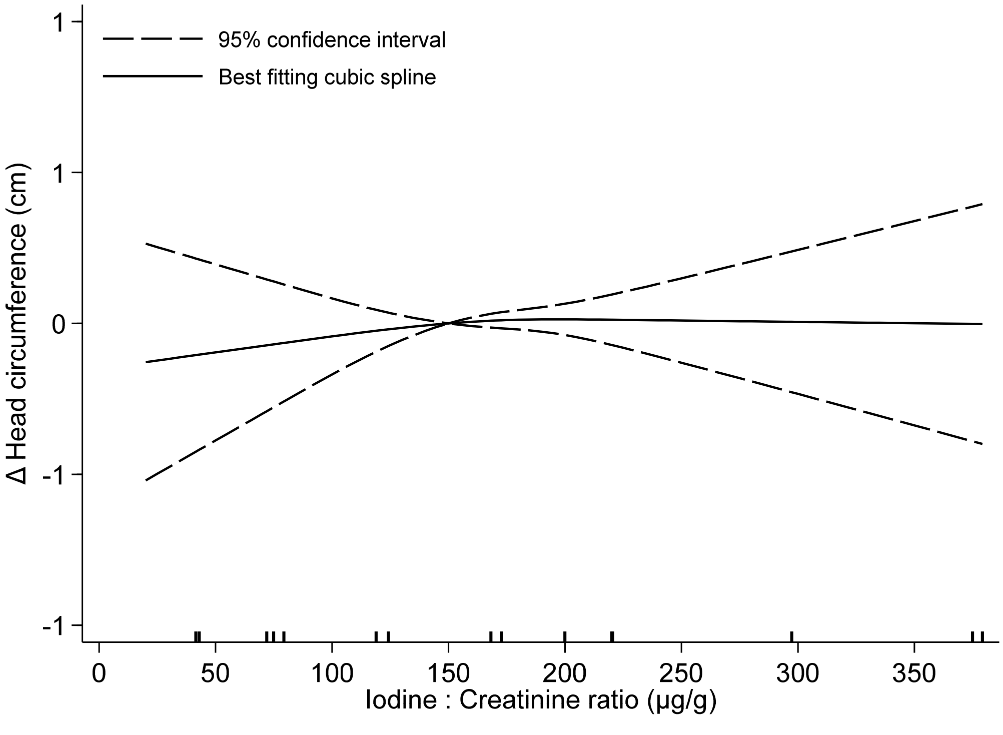

Supplementary Figure S8: Preterm delivery and iodide intake forest plot for linear trend

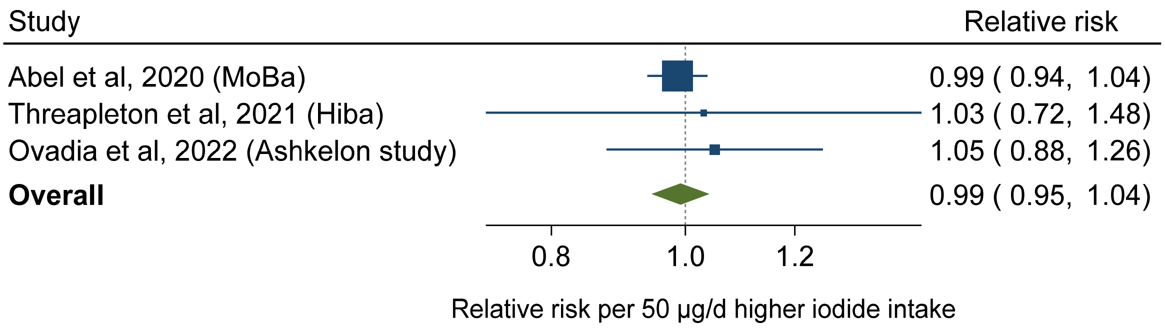

Supplementary Figure S9: Spontaneous abortion and UIC forest plot for linear trend

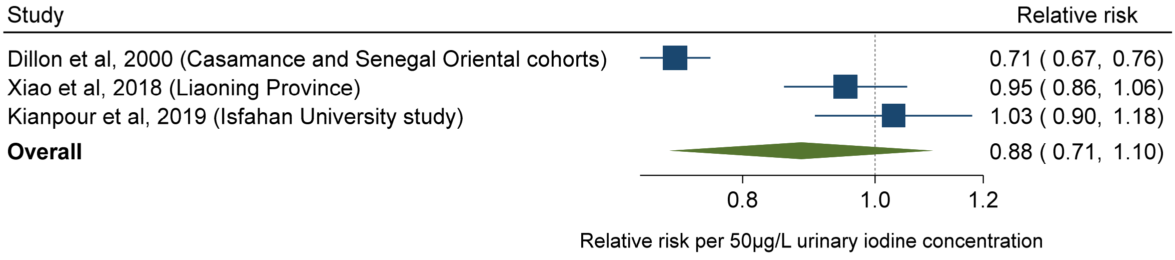

Supplementary Figure S10: Association between UIC and birth weight by mean gestation when urine sample provided

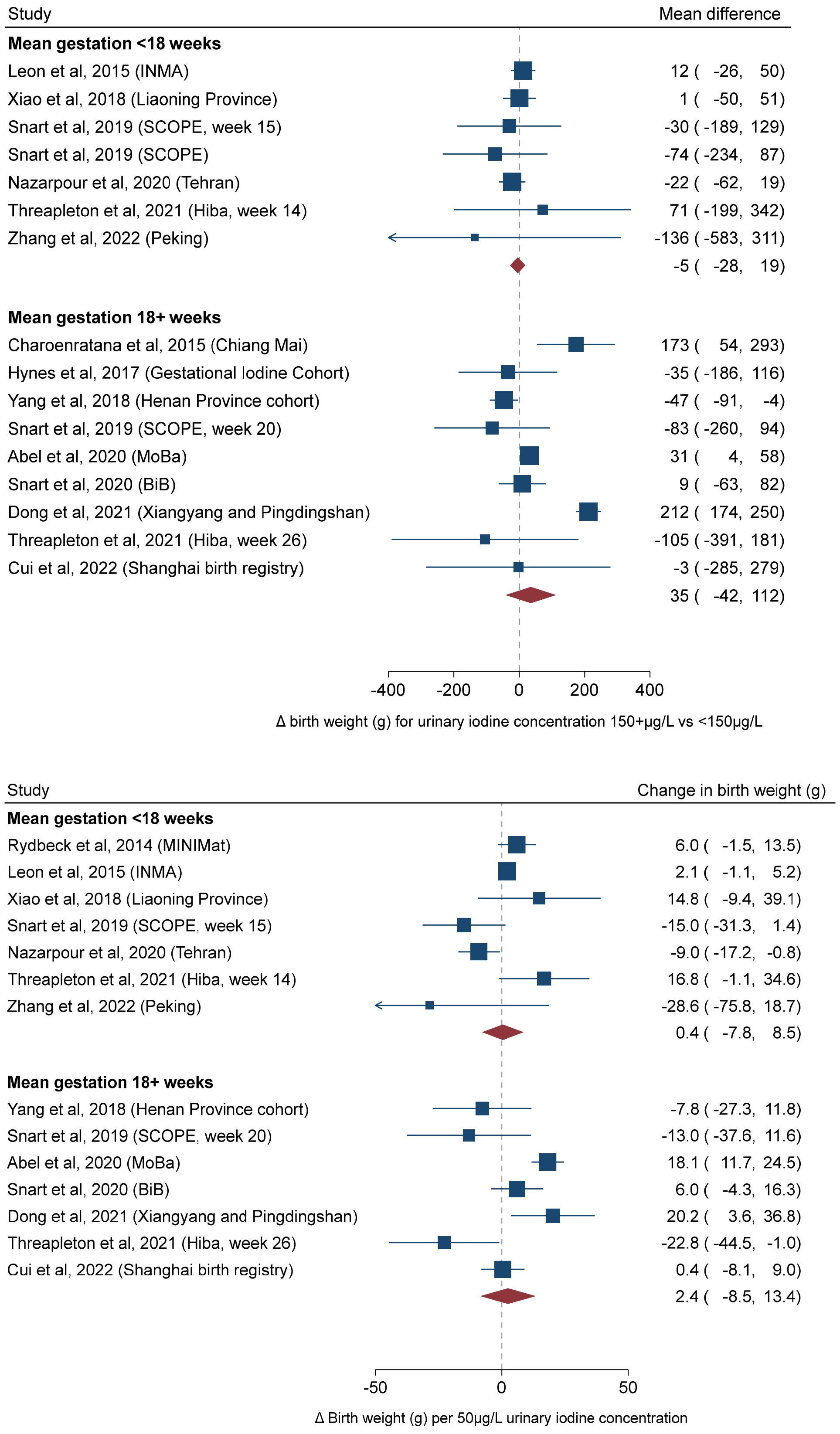

Supplementary Figure S11: Association between UIC and birth weight by median UIC of study population

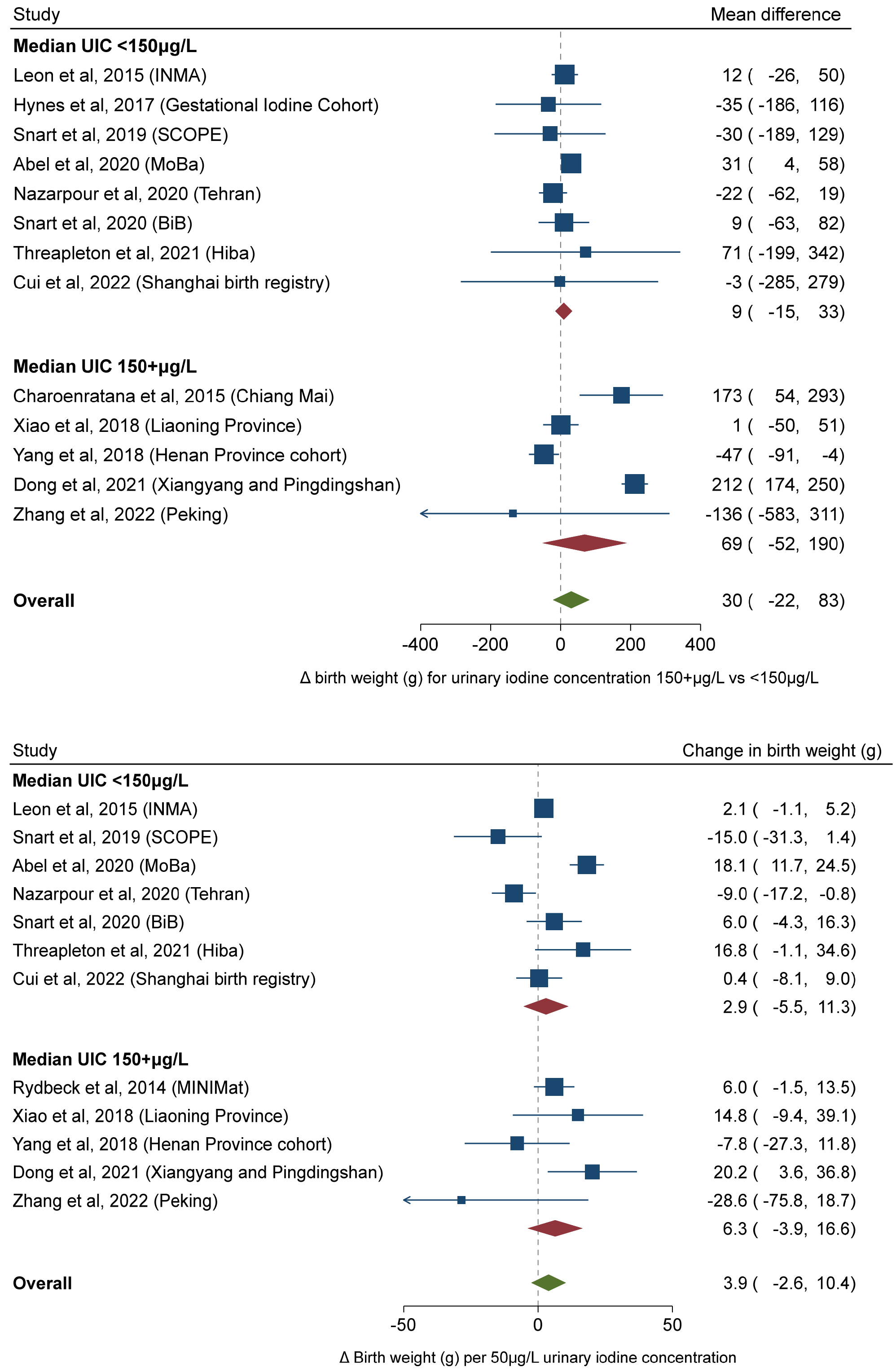

Supplementary Figure S12: Association between UIC and birth weight by income status of country

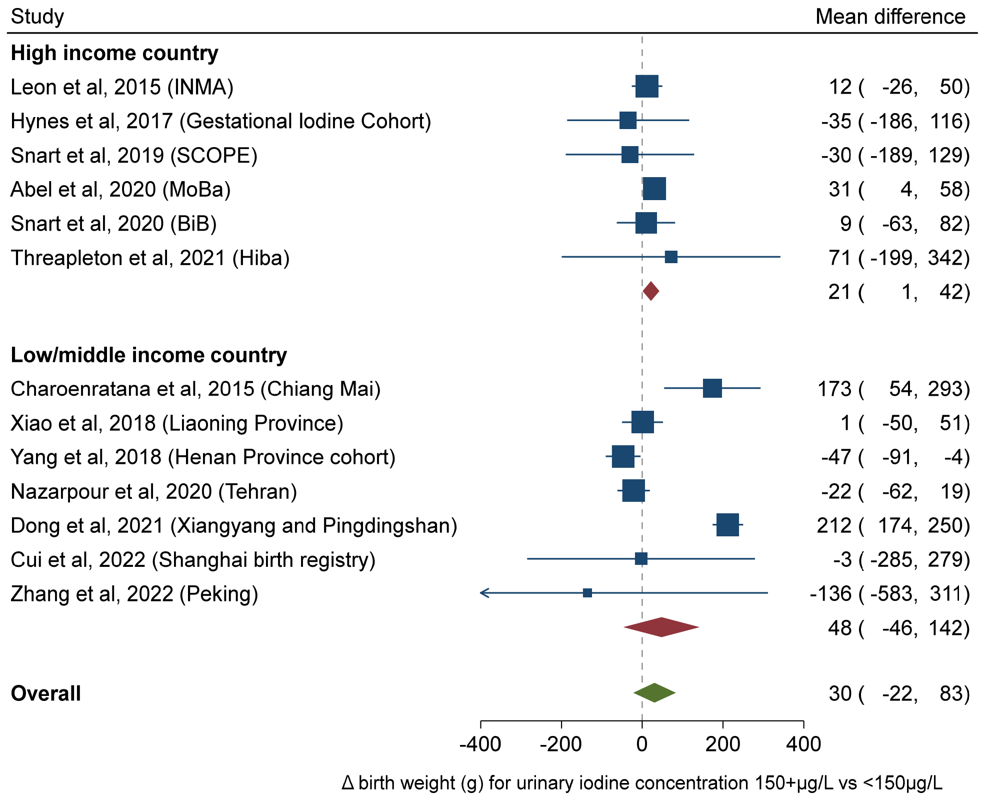

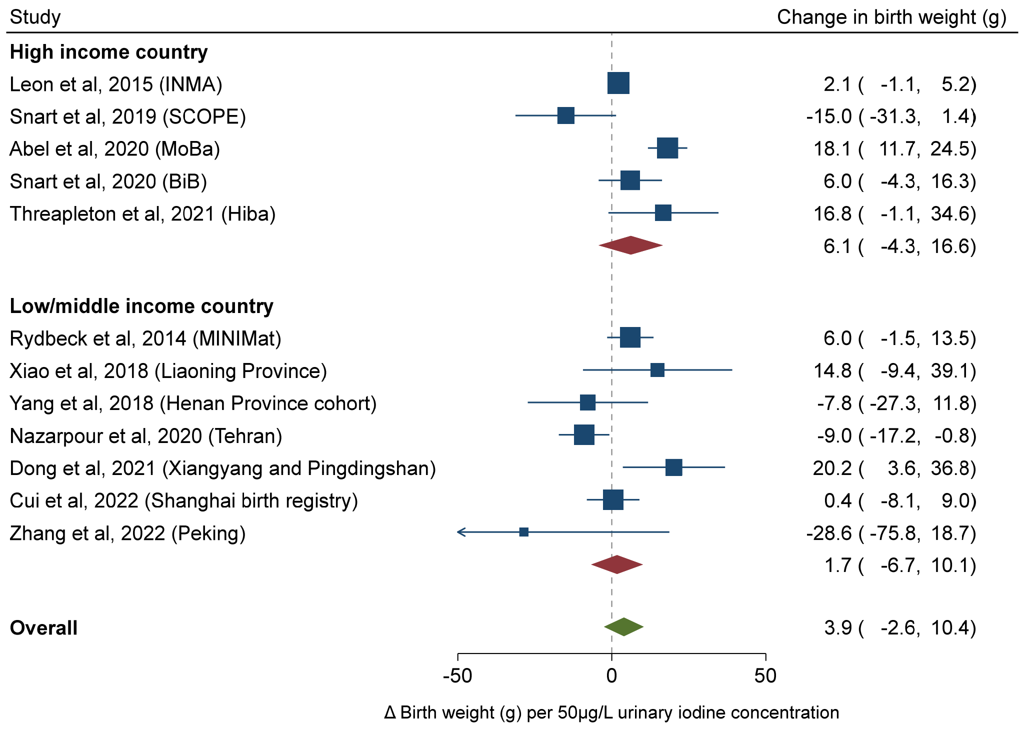

Supplementary Figure S13: Association between UIC and birth weight by adjustment for potential confounding

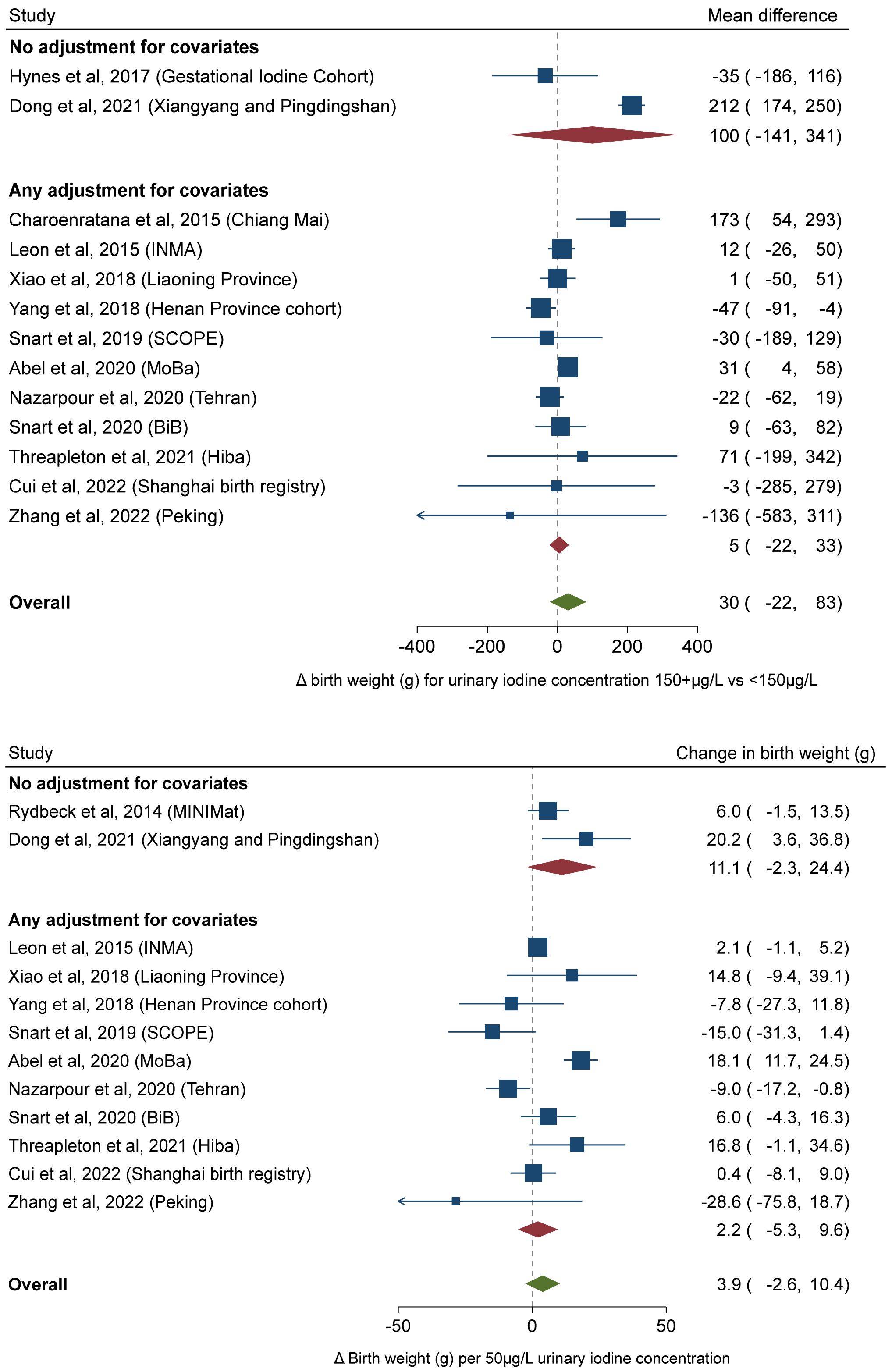

Supplementary Figure S14: Association between UIC and birth weight by Newcastle-Ottawa quality assessment score for selection

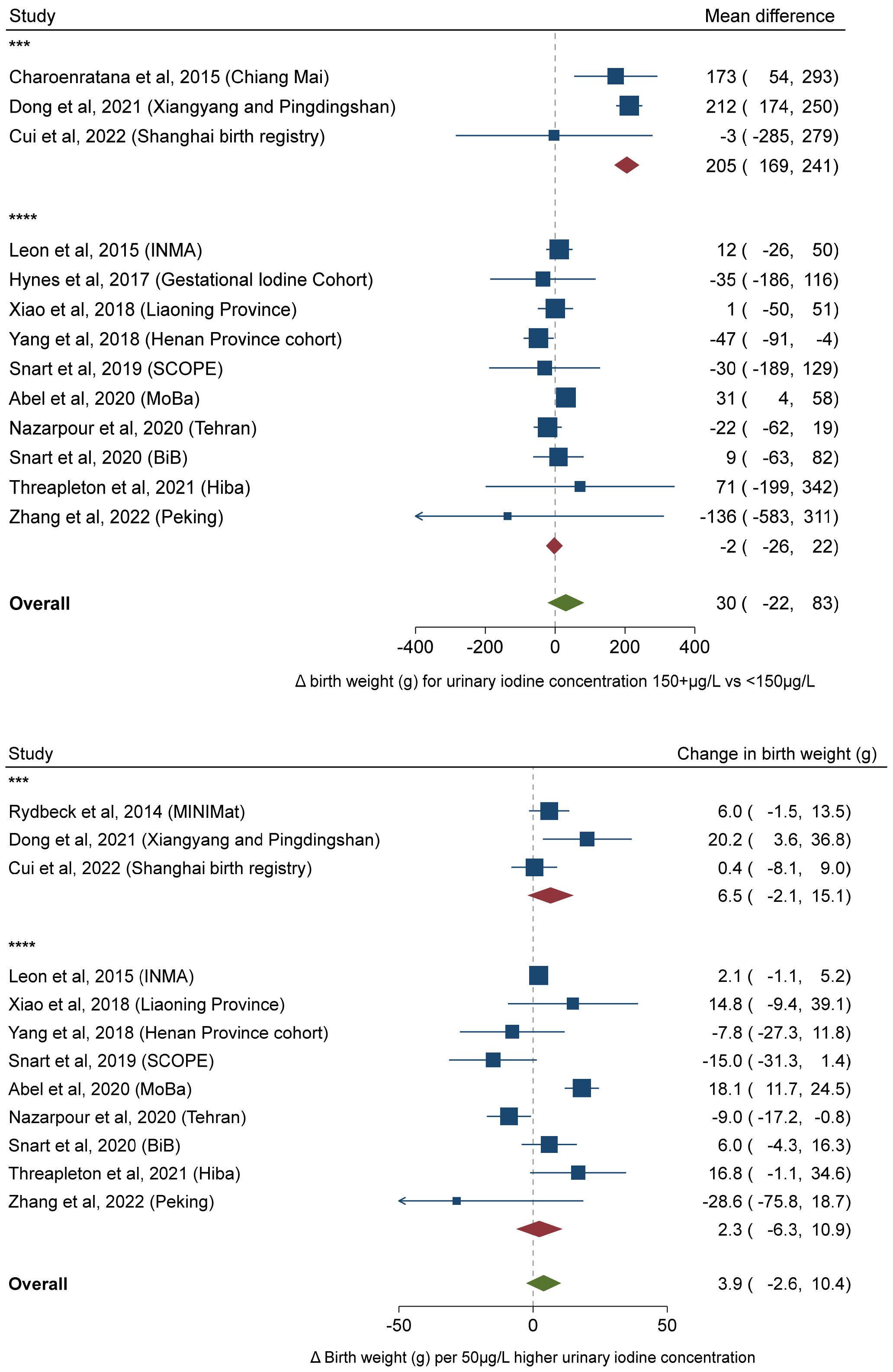

Supplementary Figure S15: Association between UIC and birth weight by Newcastle-Ottawa quality assessment score for comparability

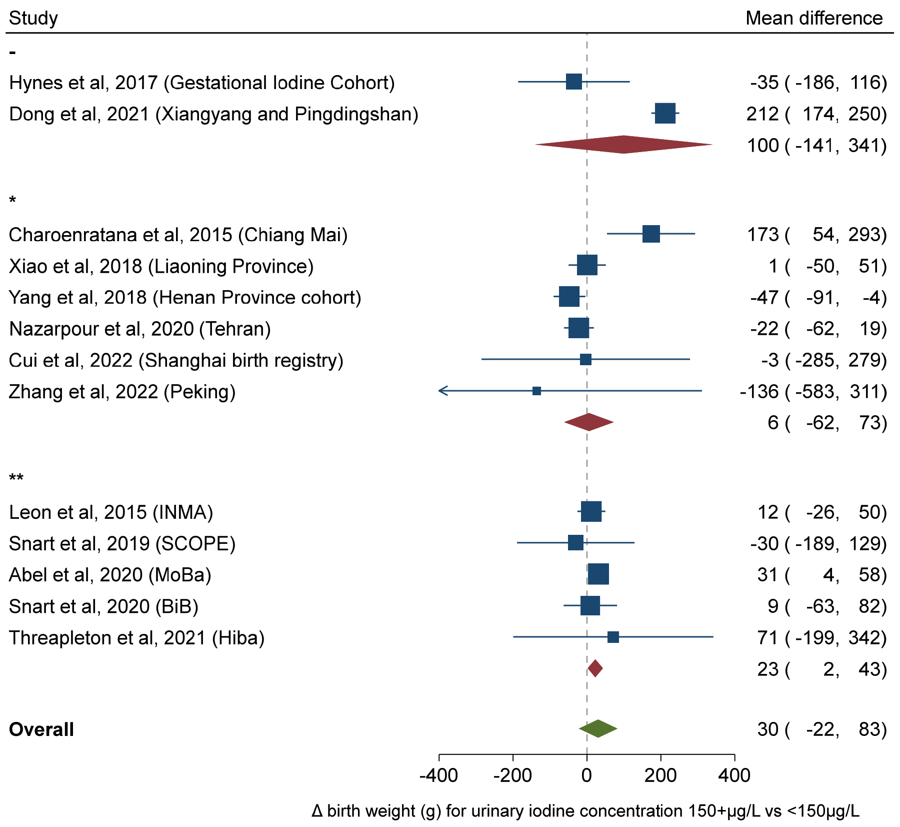

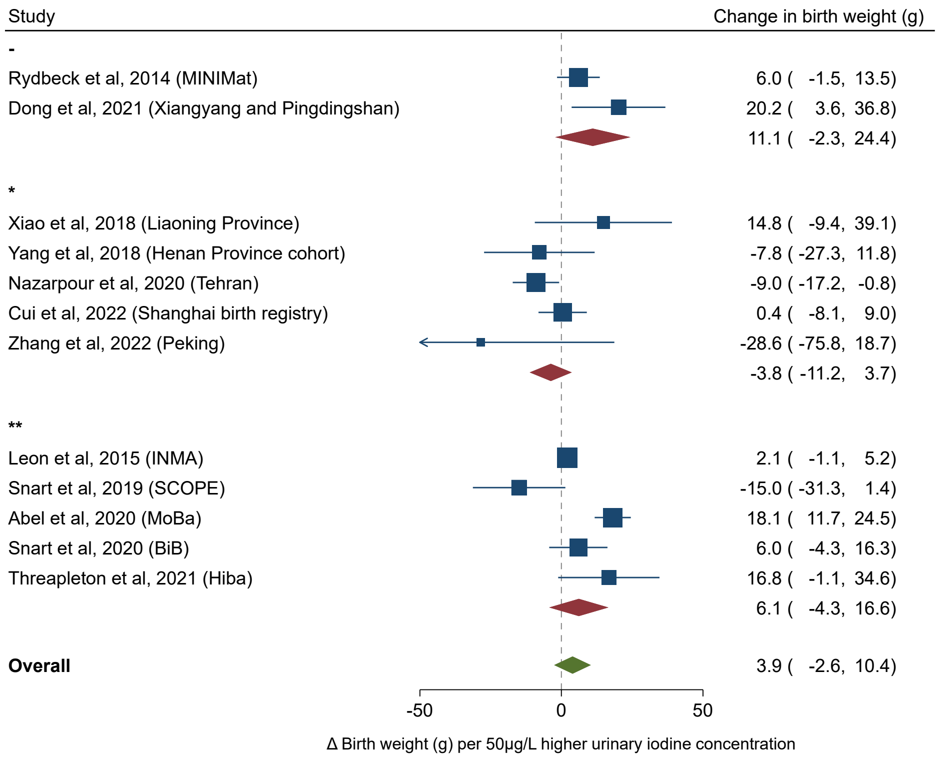

Supplementary Figure S16: Association between UIC and birth weight by Newcastle-Ottawa quality assessment score for outcome

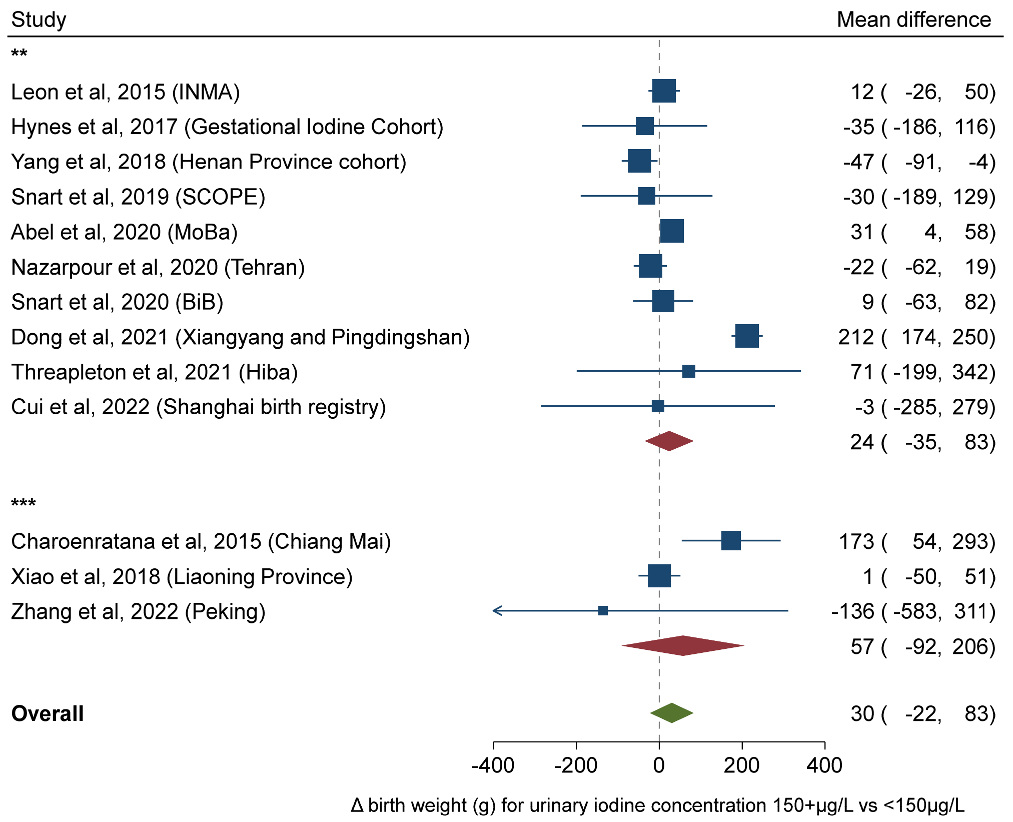

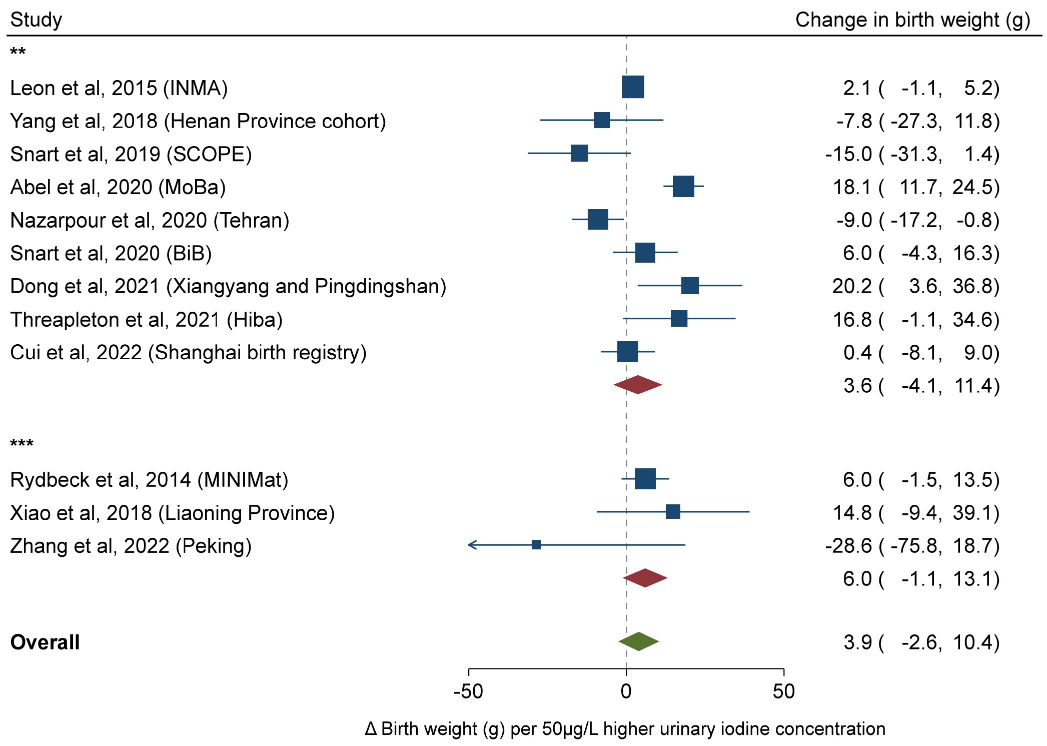

Supplementary Figure S17: Association between UIC and SGA by mean gestation when urine sample provided

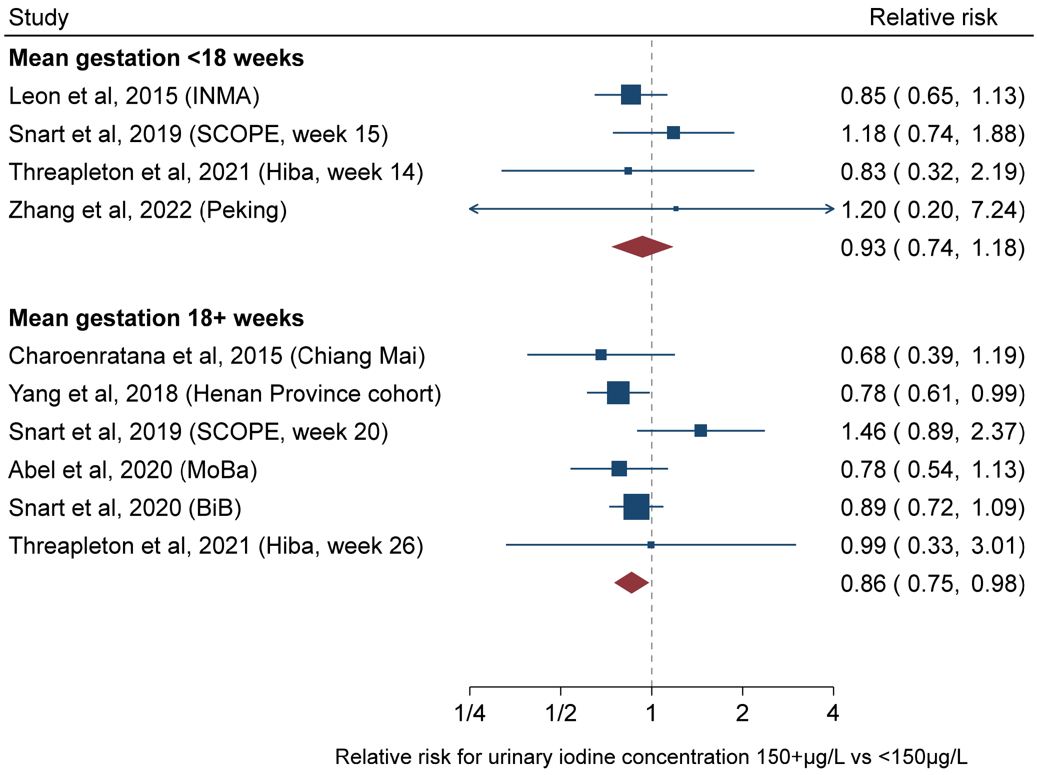

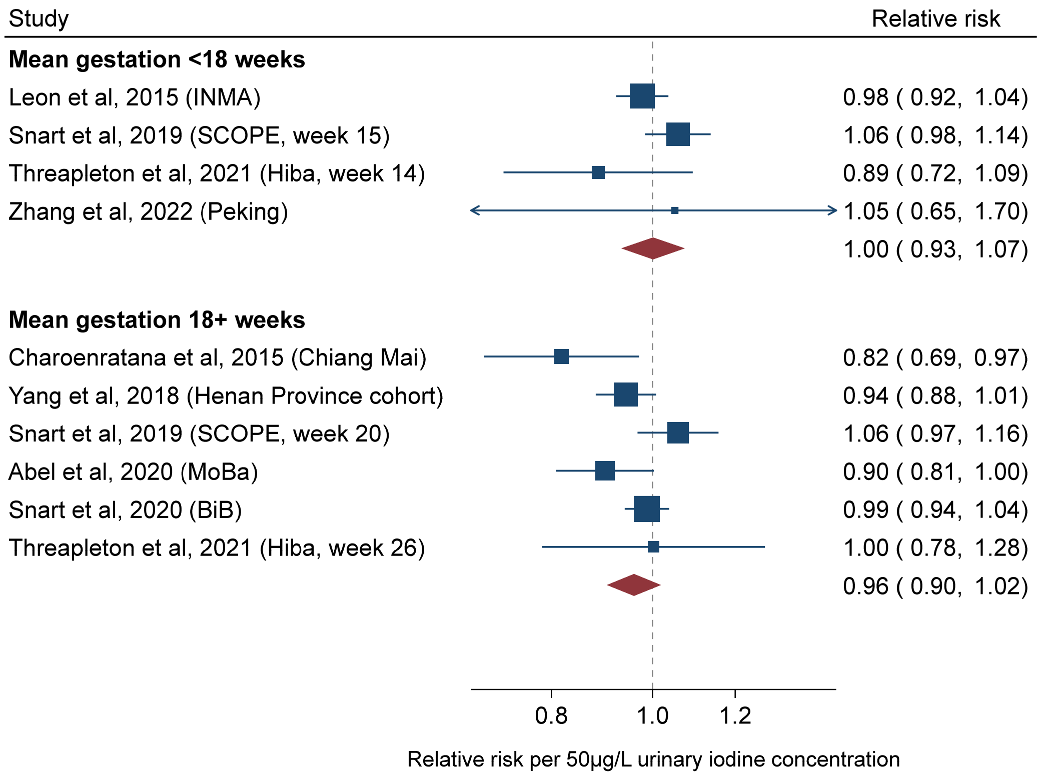

Supplementary Figure S18: Association between UIC and SGA by median UIC of study population

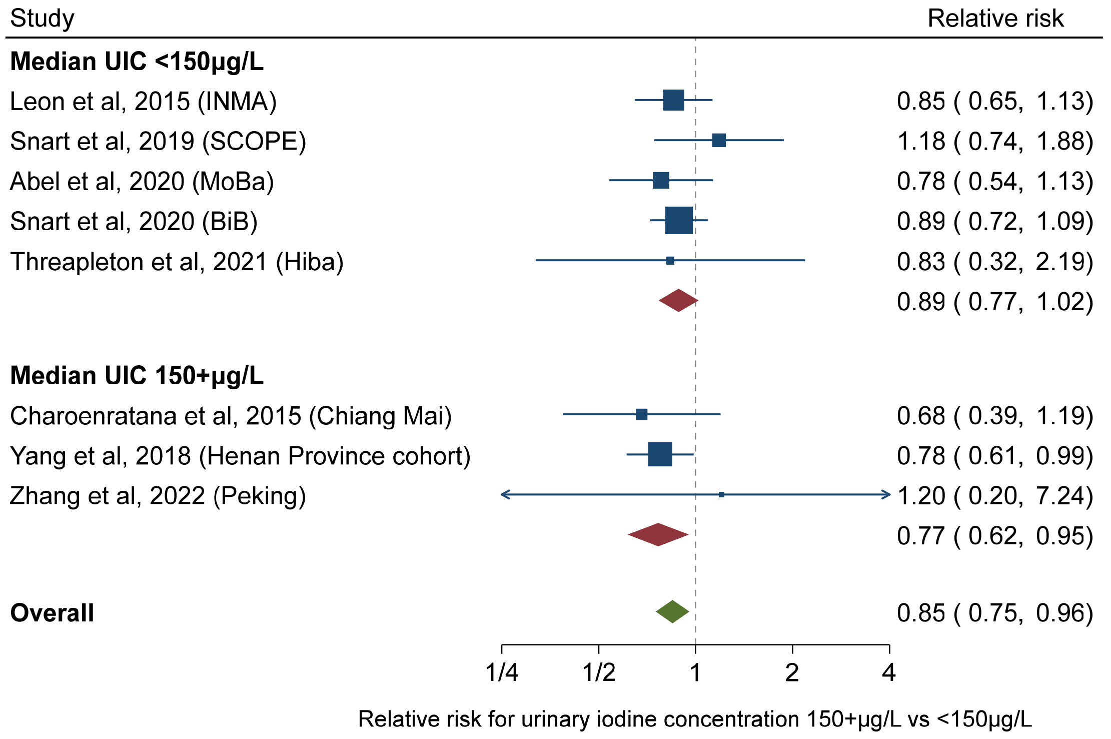

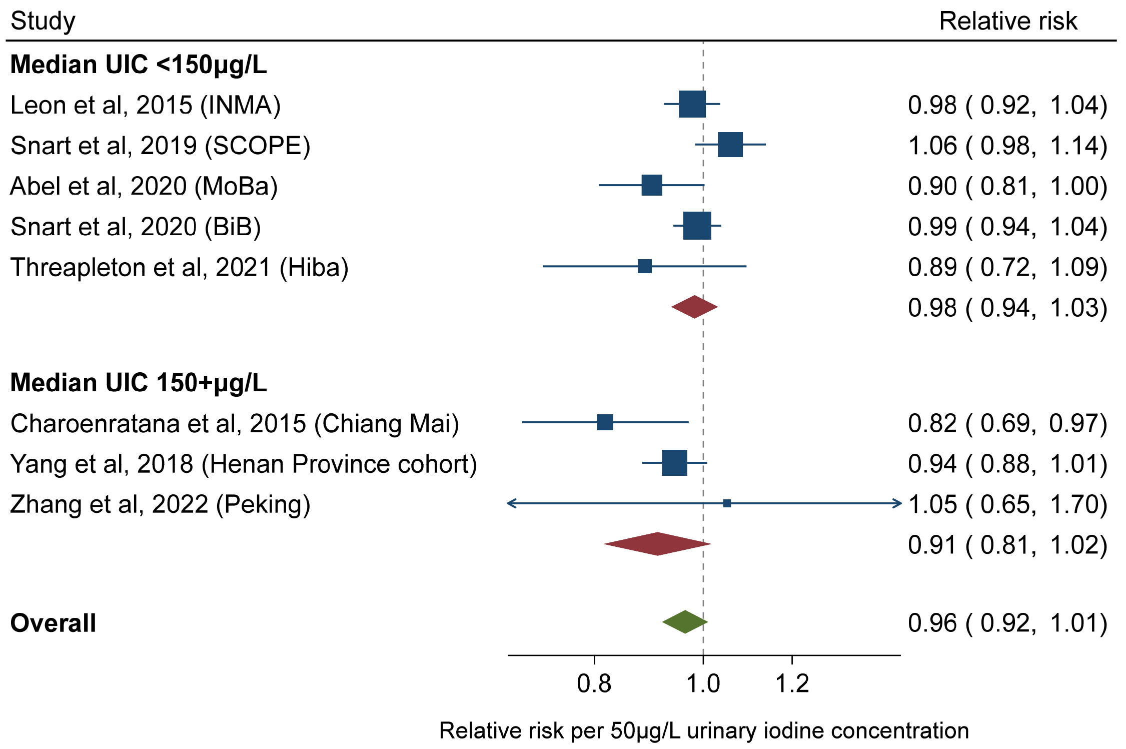

Supplementary Figure S19: Association between UIC and SGA by income status of country

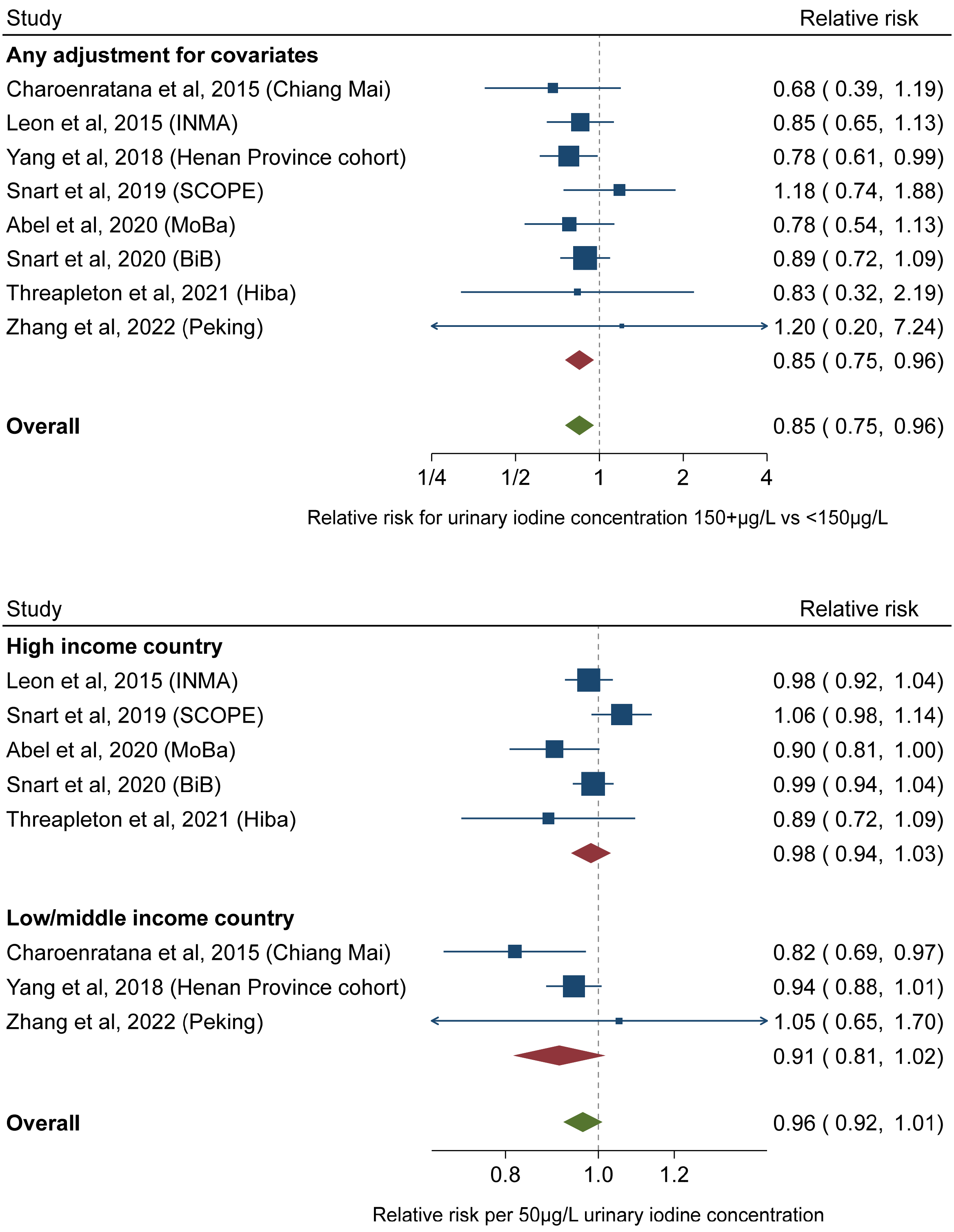

Supplementary Figure S20: Association between UIC and SGA by adjustment for potential confounding

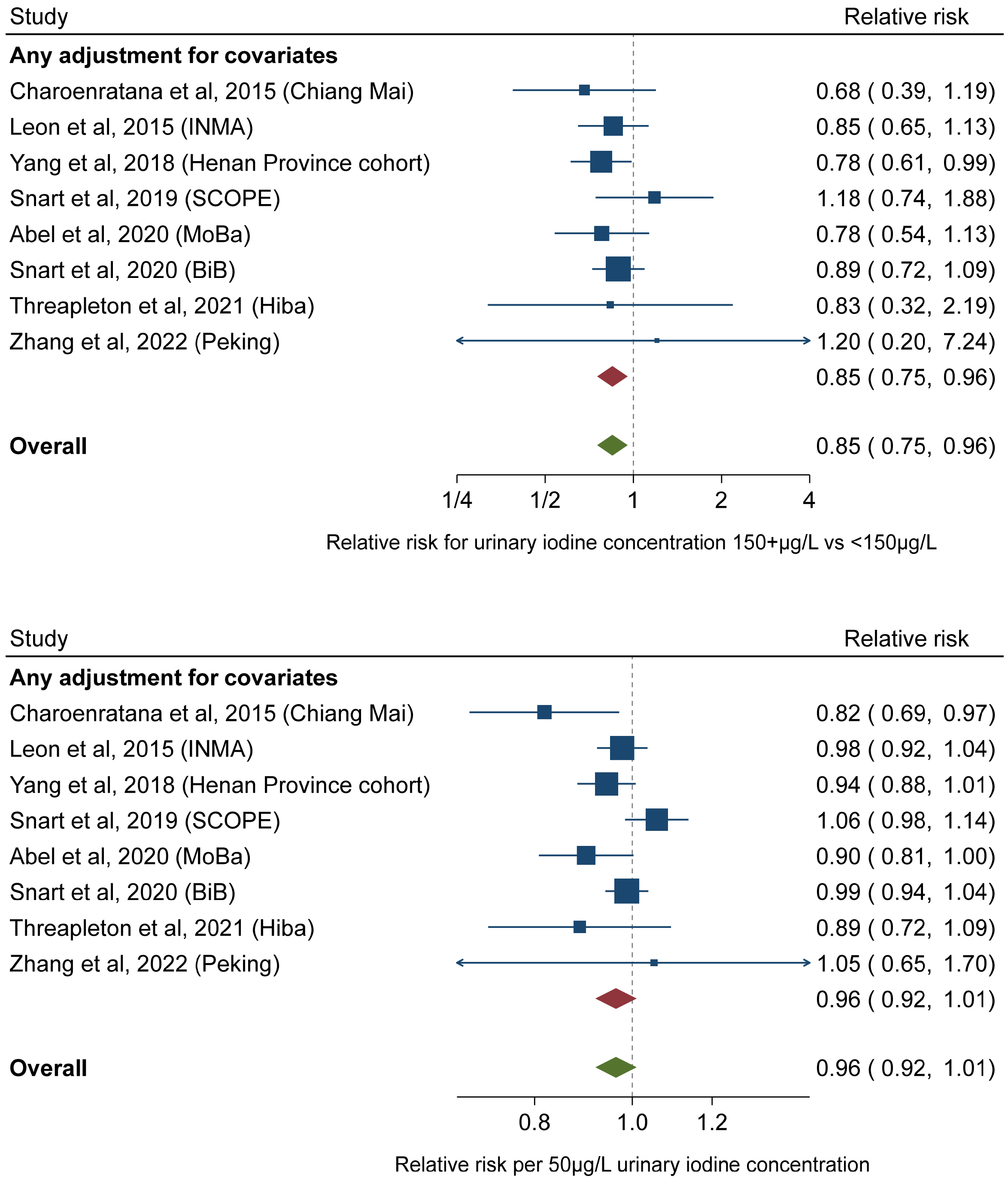

Supplementary Figure S21: Association between UIC and SGA by Newcastle-Ottawa quality assessment score for selection

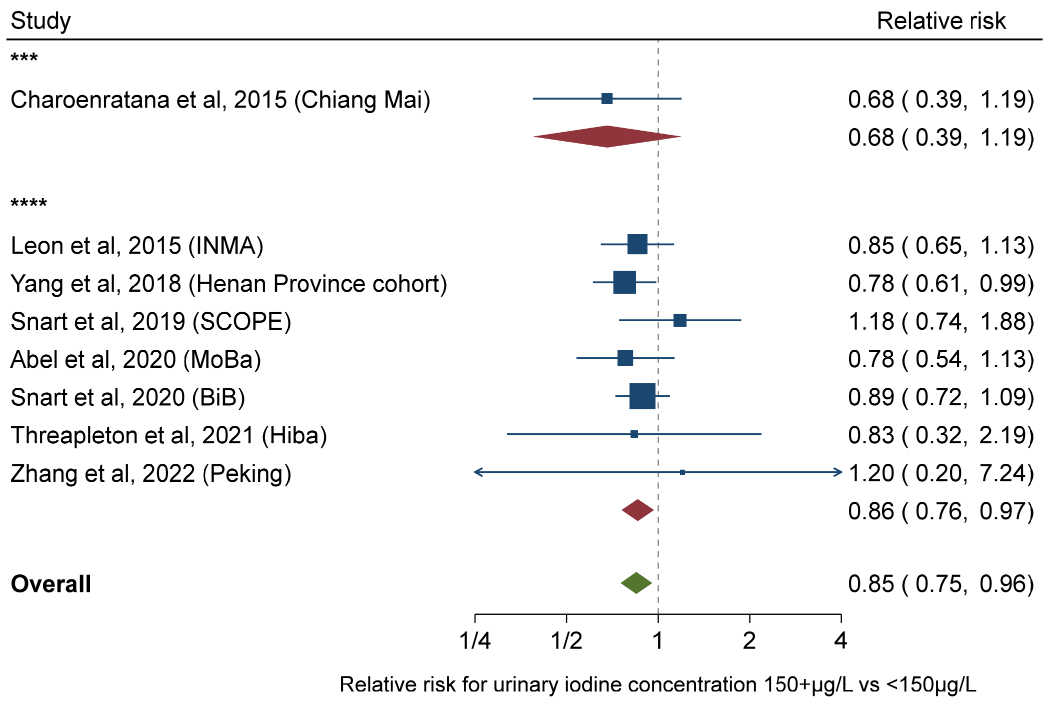

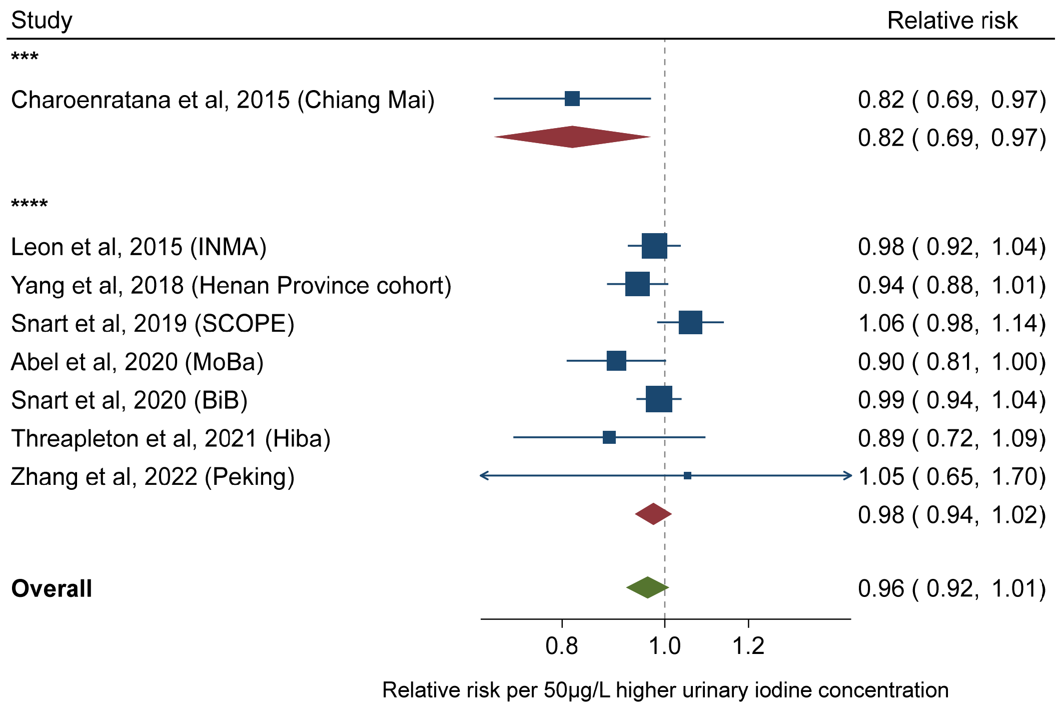

Supplementary Figure S22: Association between UIC and SGA by Newcastle-Ottawa quality assessment score for comparability

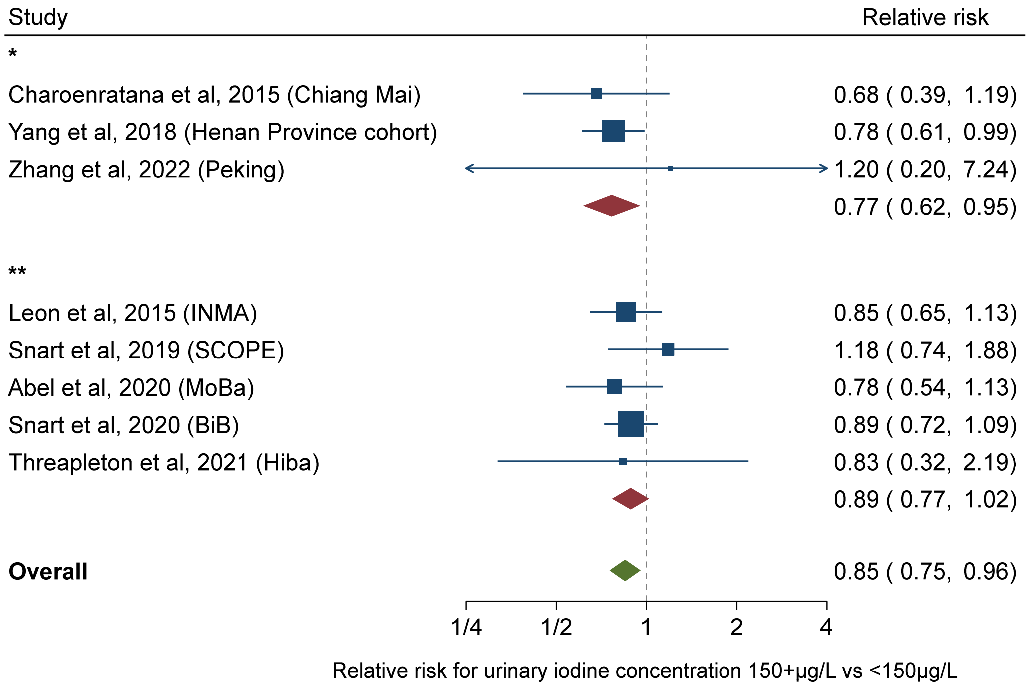

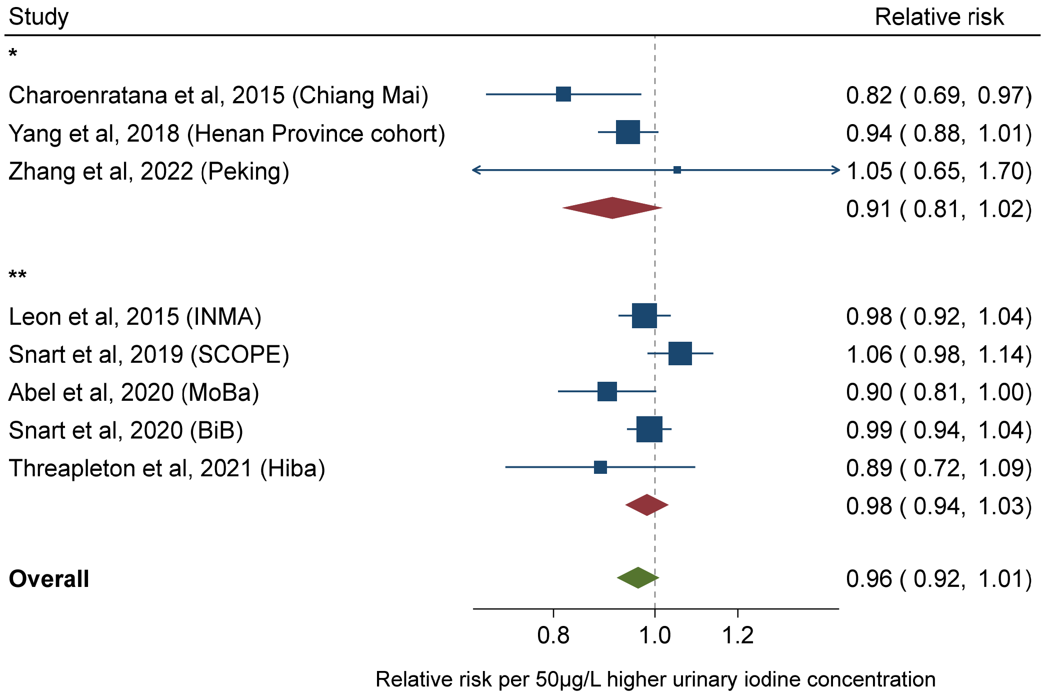

Supplementary Figure S23: Association between UIC and SGA by Newcastle-Ottawa quality assessment score for outcome

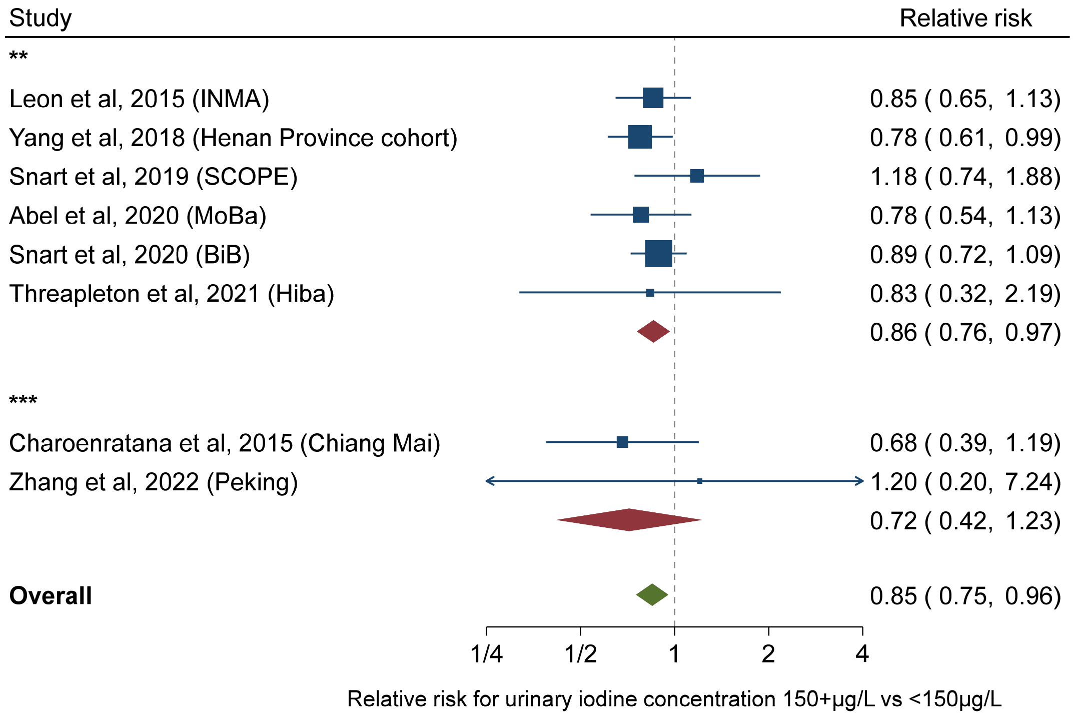

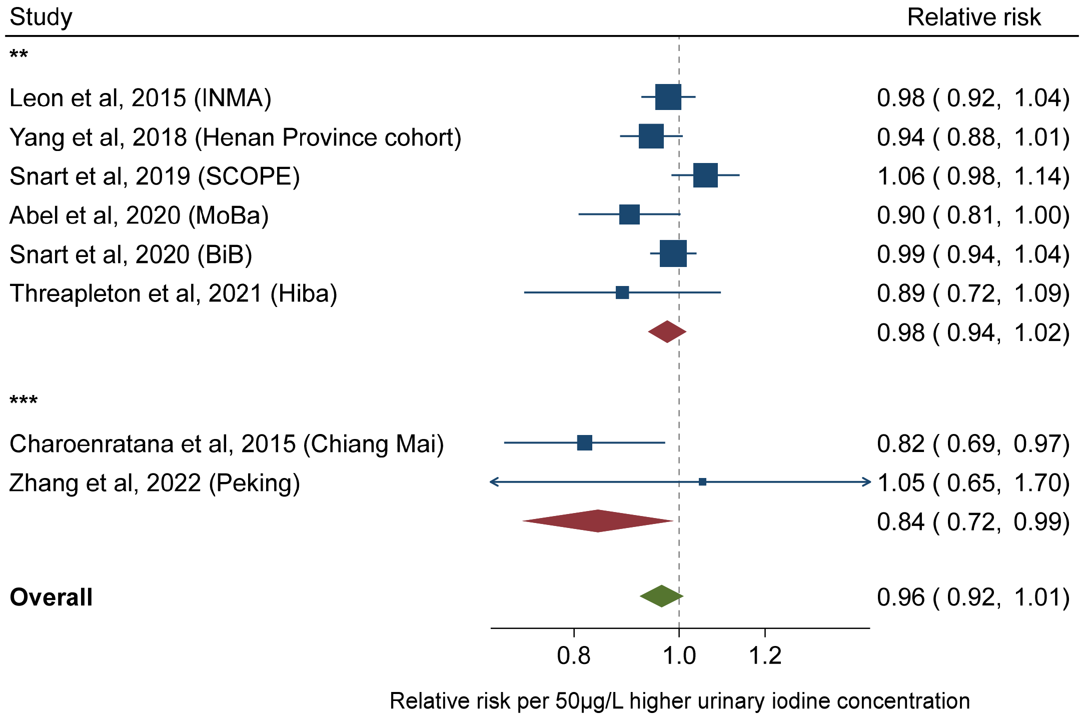

Supplementary Figure S24: Association between UIC and preterm delivery by mean gestation when urine sample provided

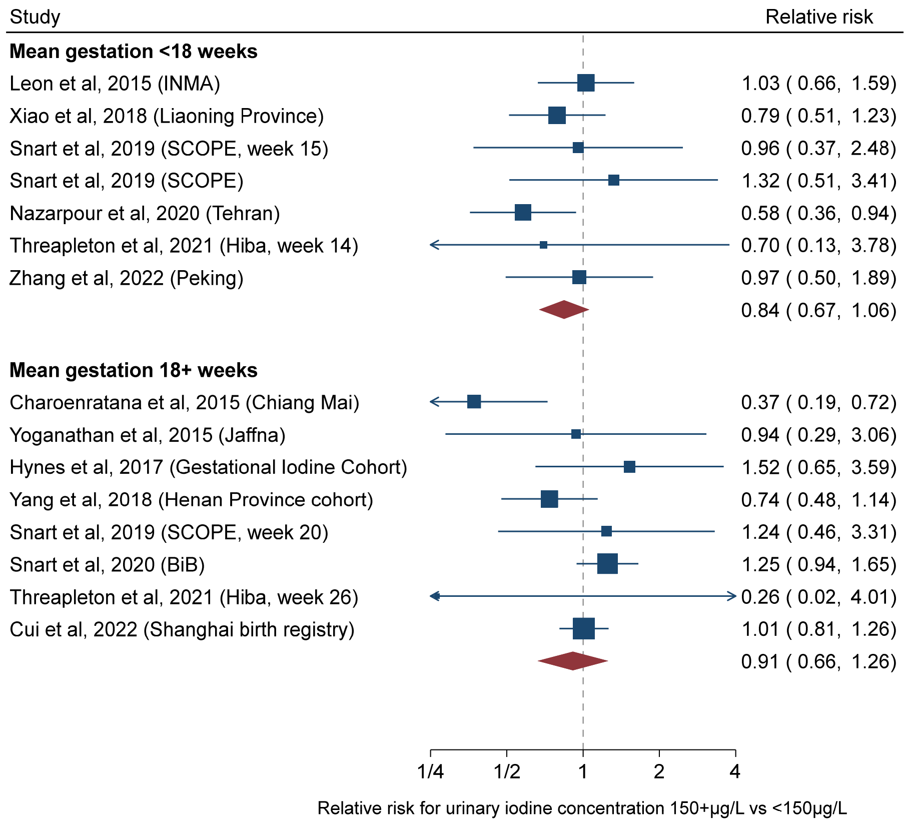

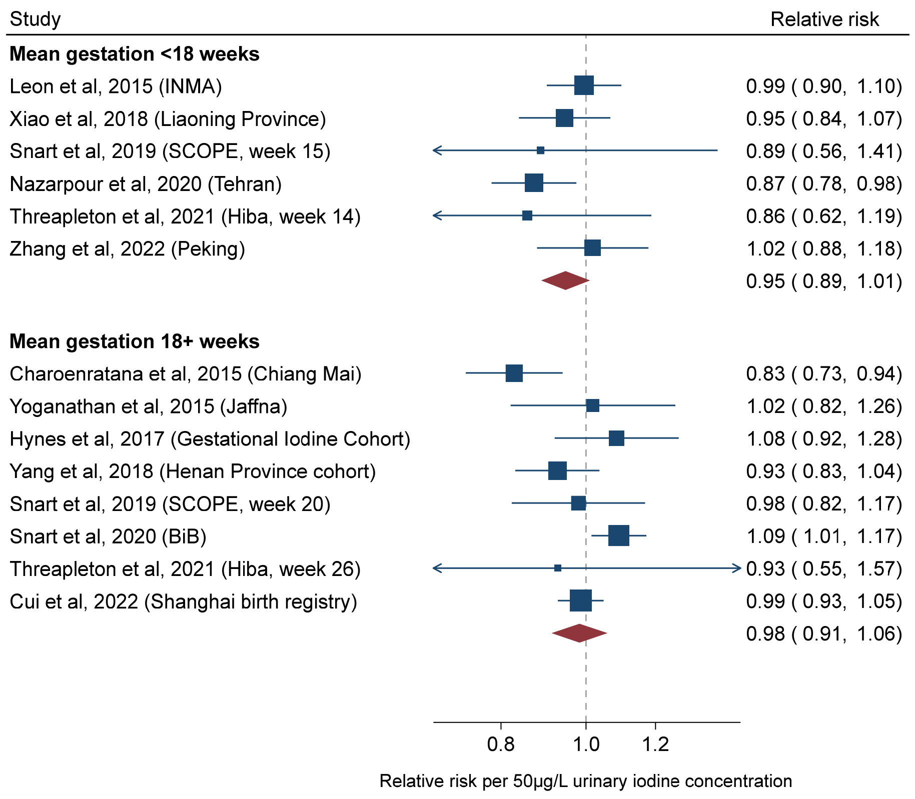

Supplementary Figure S25: Association between UIC and preterm delivery by median UIC of study population

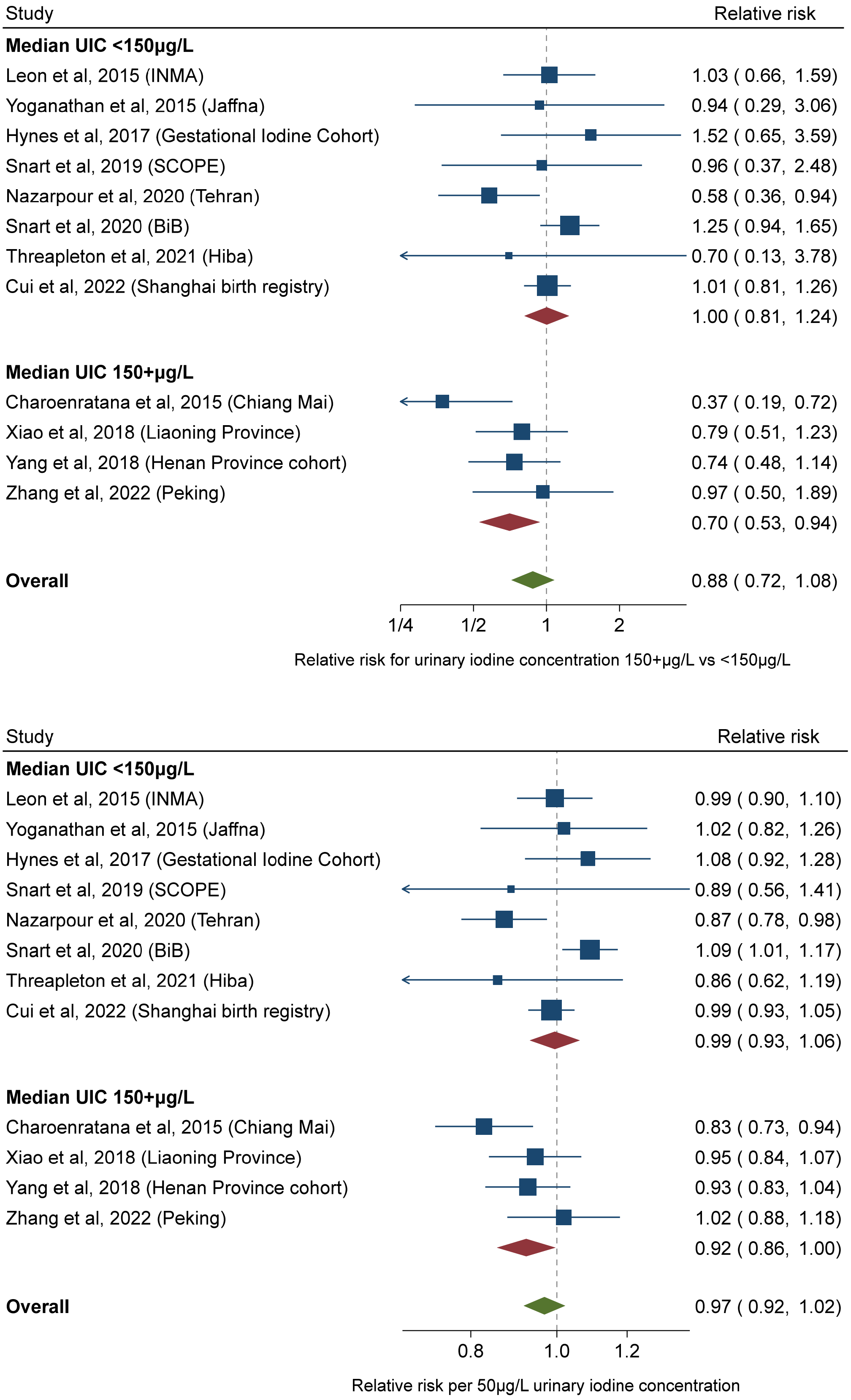

Supplementary Figure S26: Association between UIC and preterm delivery by income status of country

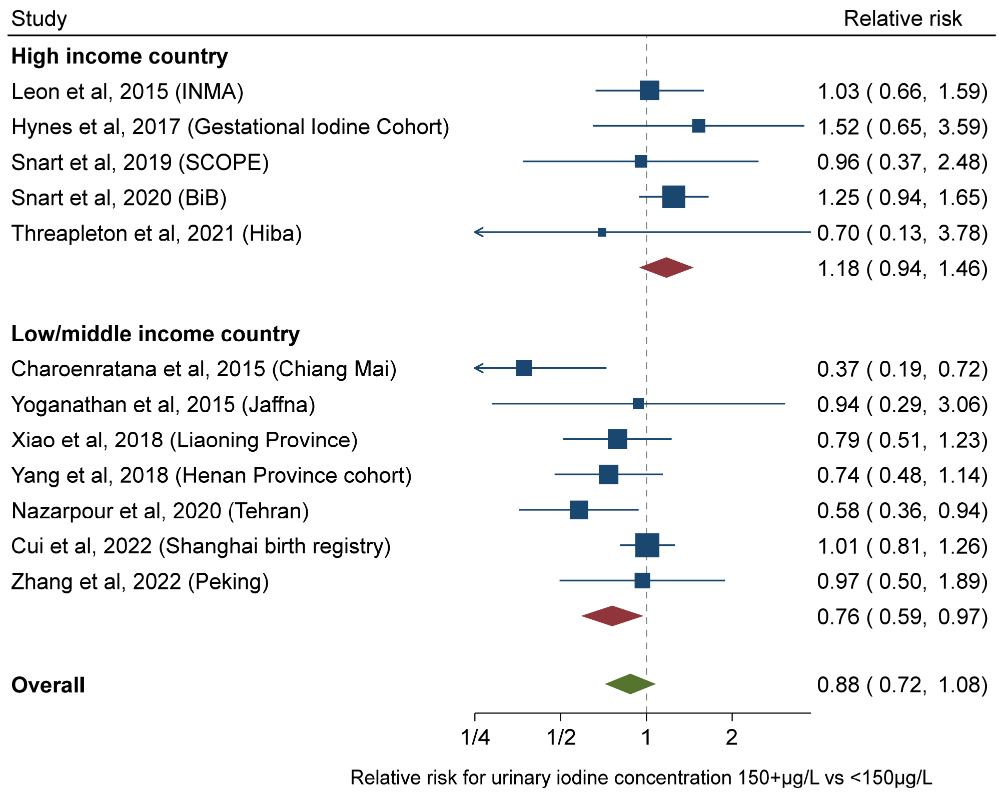

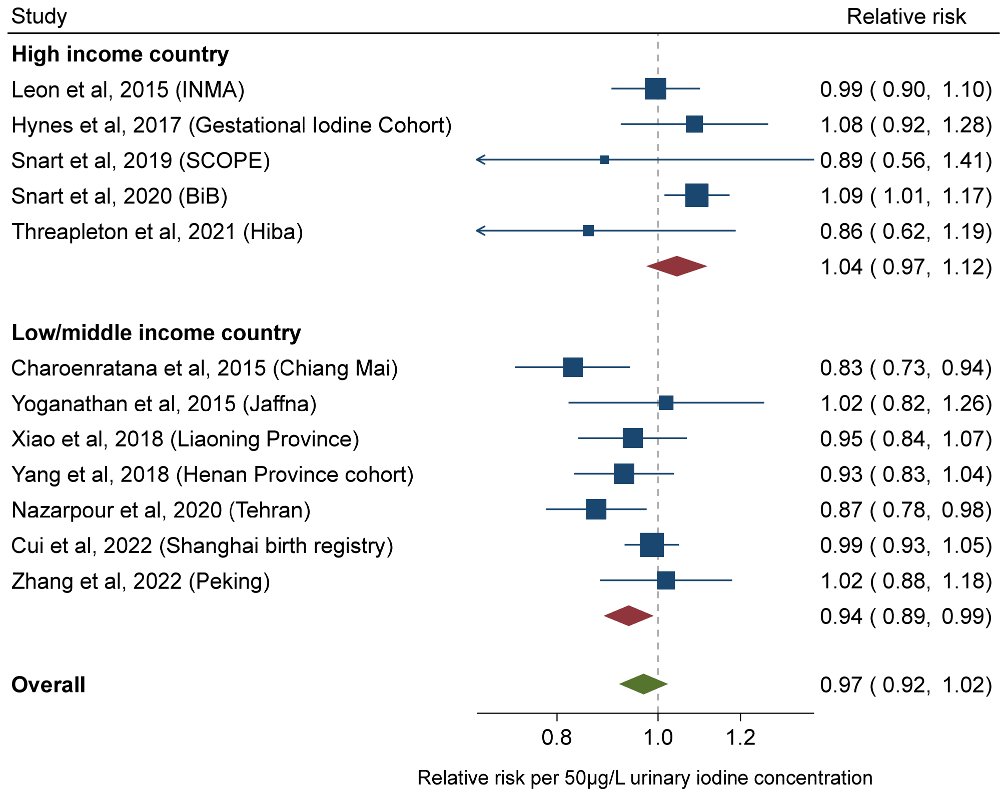

Supplementary Figure S27: Association between UIC and preterm delivery by adjustment for potential confounding

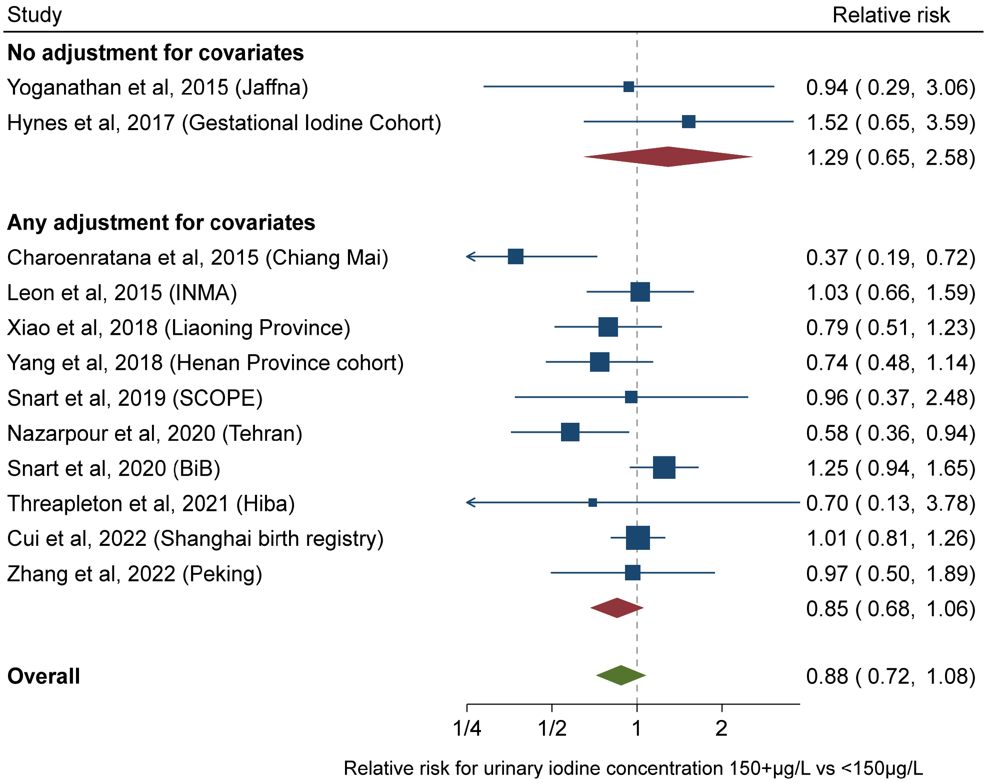

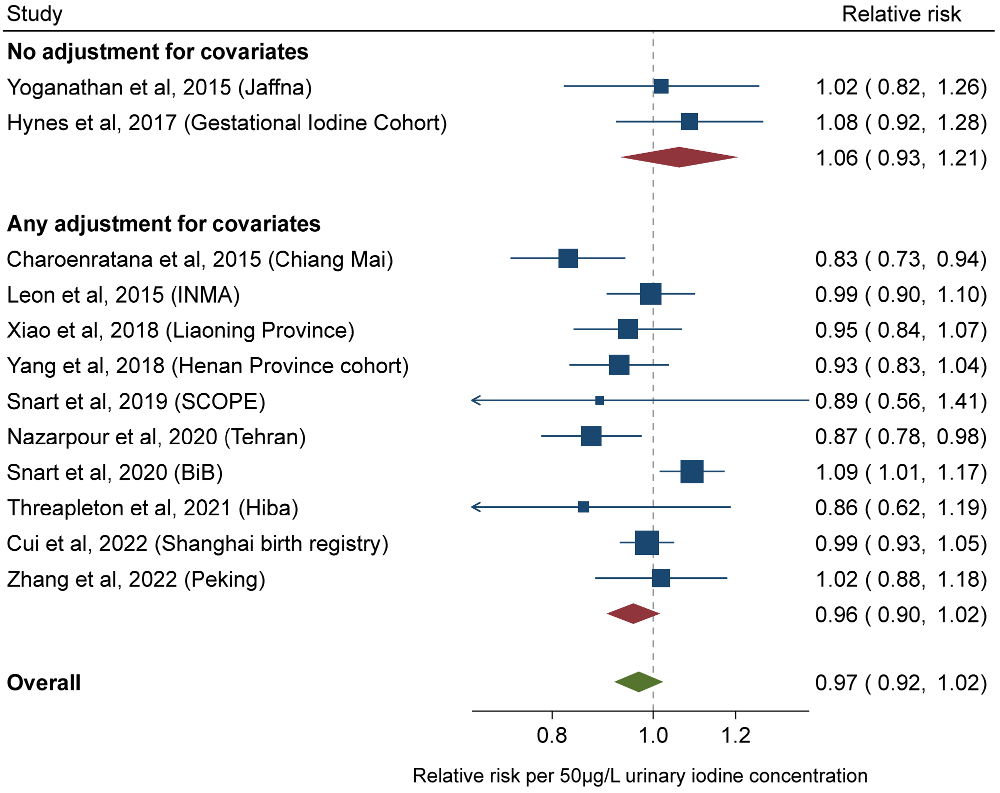

Supplementary Figure S28: Association between UIC and preterm delivery by Newcastle-Ottawa quality assessment score for selection

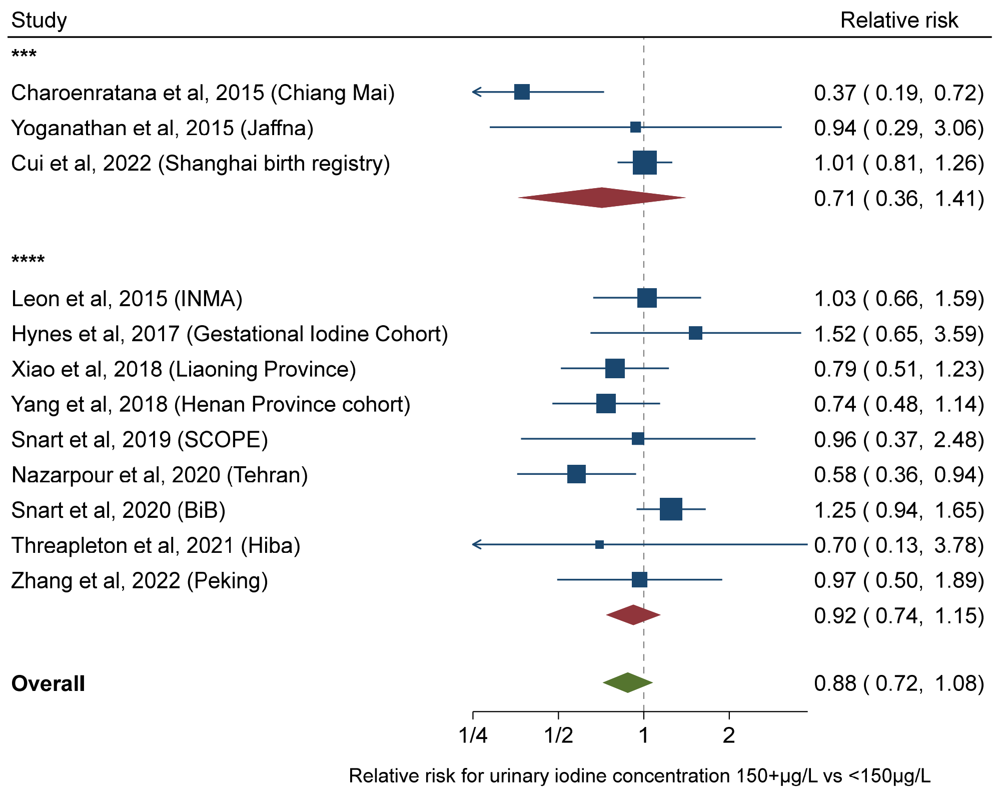

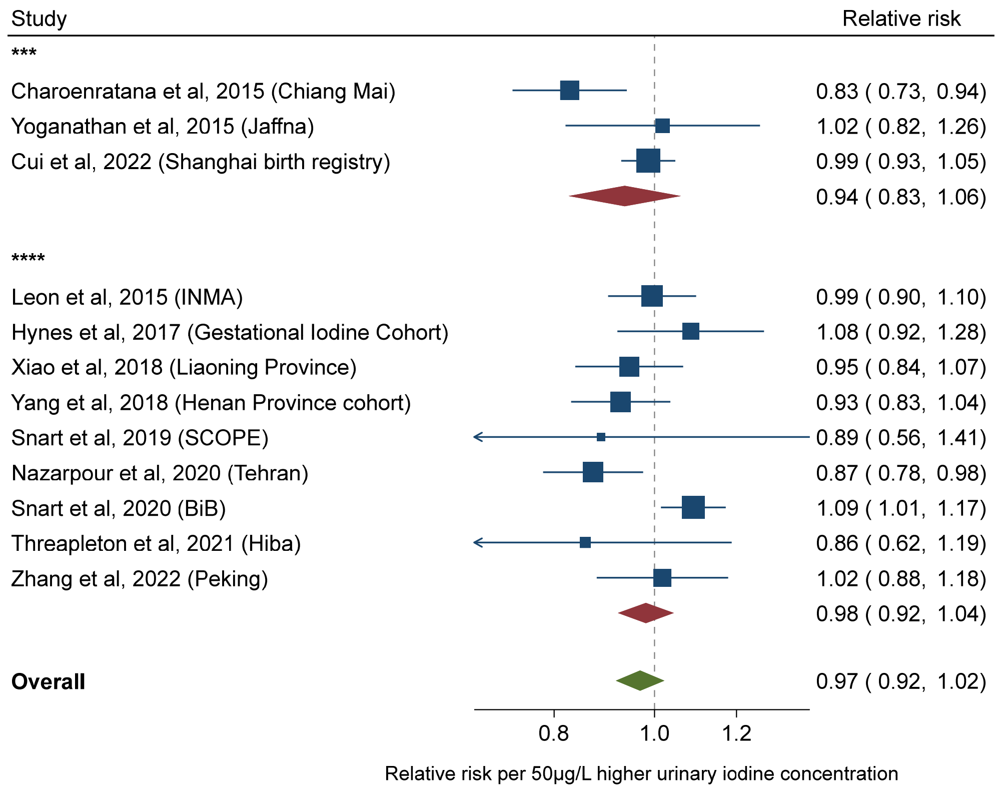

Supplementary Figure S29: Association between UIC and preterm delivery by Newcastle-Ottawa quality assessment score for comparability

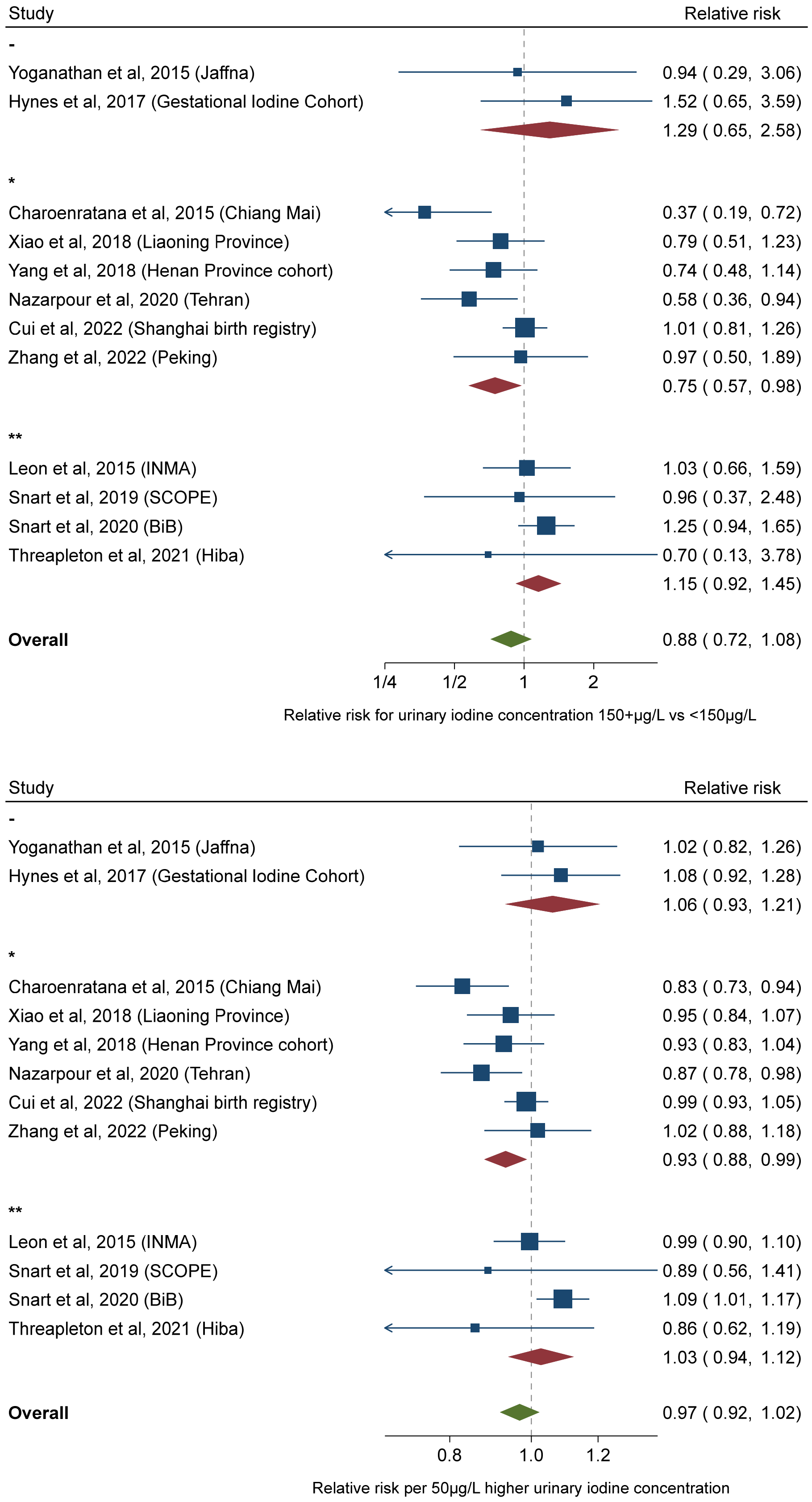

Supplementary Figure S30: Association between UIC and preterm delivery by Newcastle-Ottawa quality assessment score for outcome

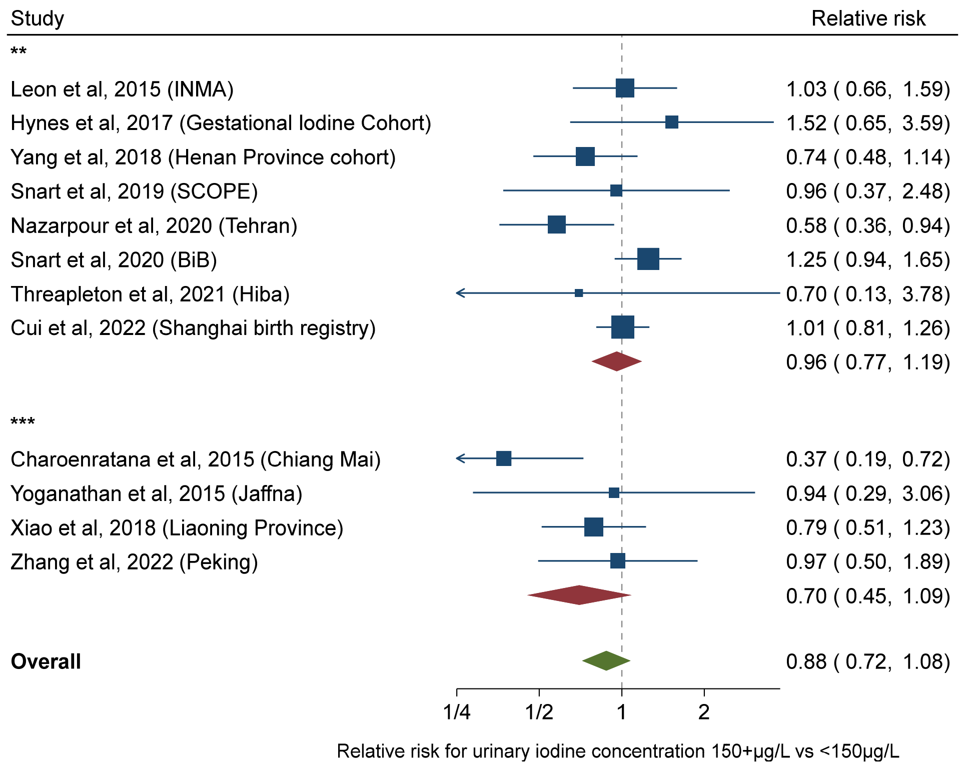

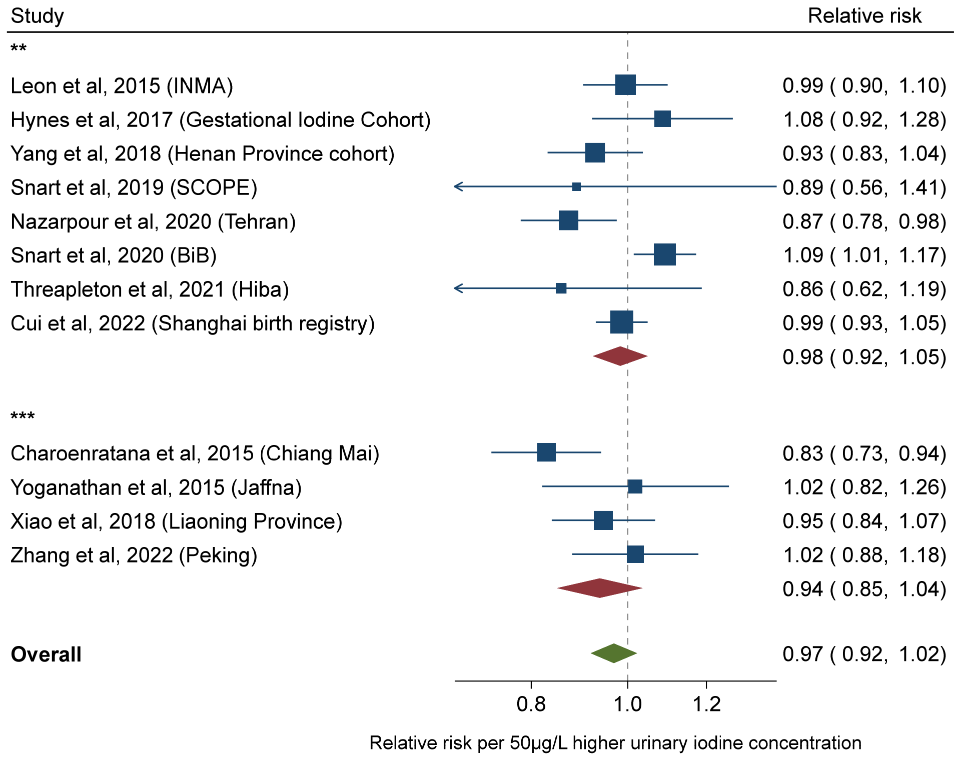

Supplementary Figure S31: Contour-enhanced funnel plots for UIC and birth weight

Meta-analysis of dichotomised UIC ≥150µg/L vs UIC<150 µg/L

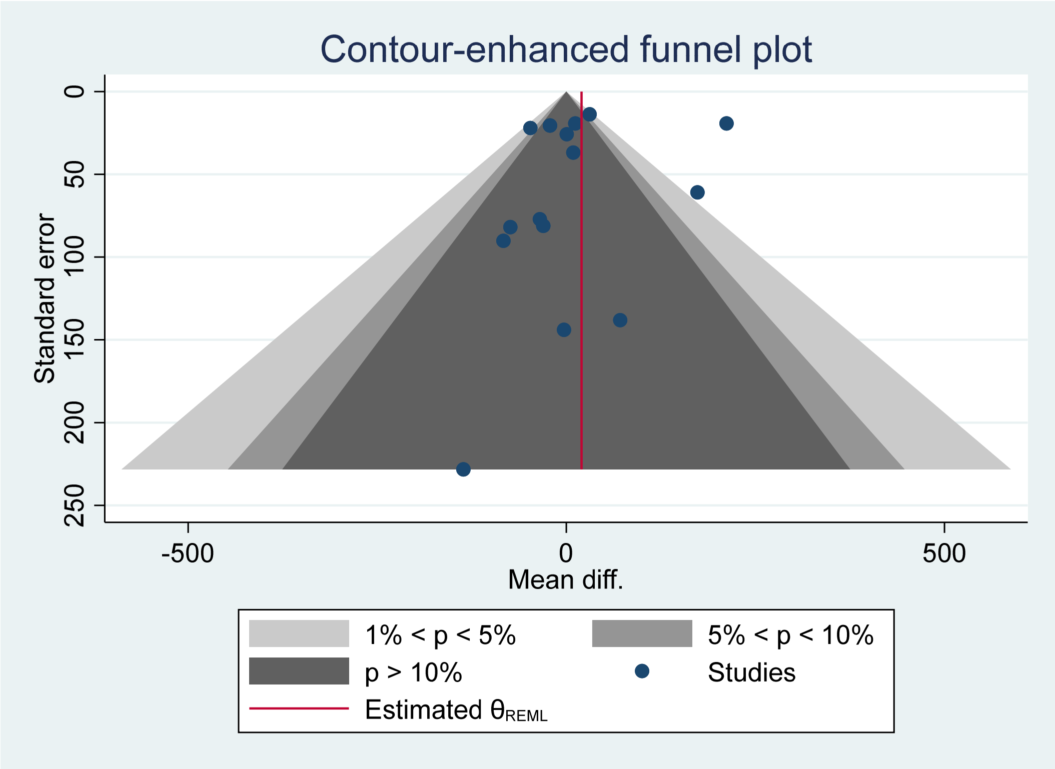

Meta-analysis of linear trend per 50µg/L UIC

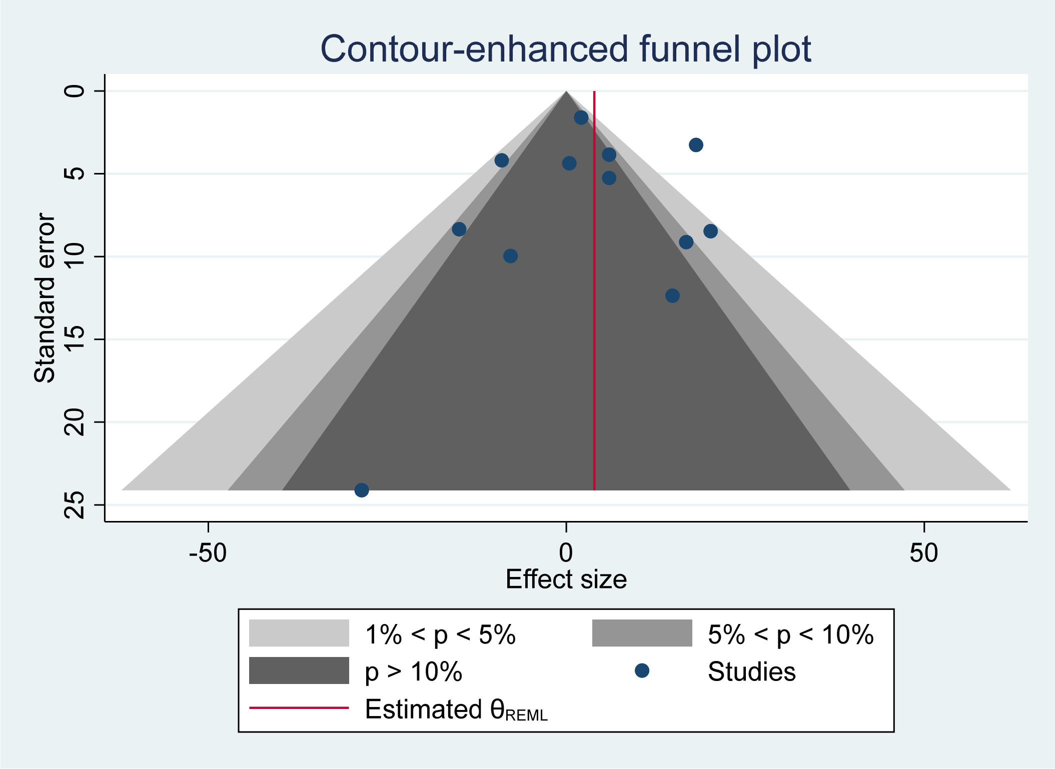

Supplementary Figure S32: Contour-enhanced funnel plots for UIC and preterm delivery

Meta-analysis of dichotomised UIC ≥150μg/L vs UIC<150 μg/L

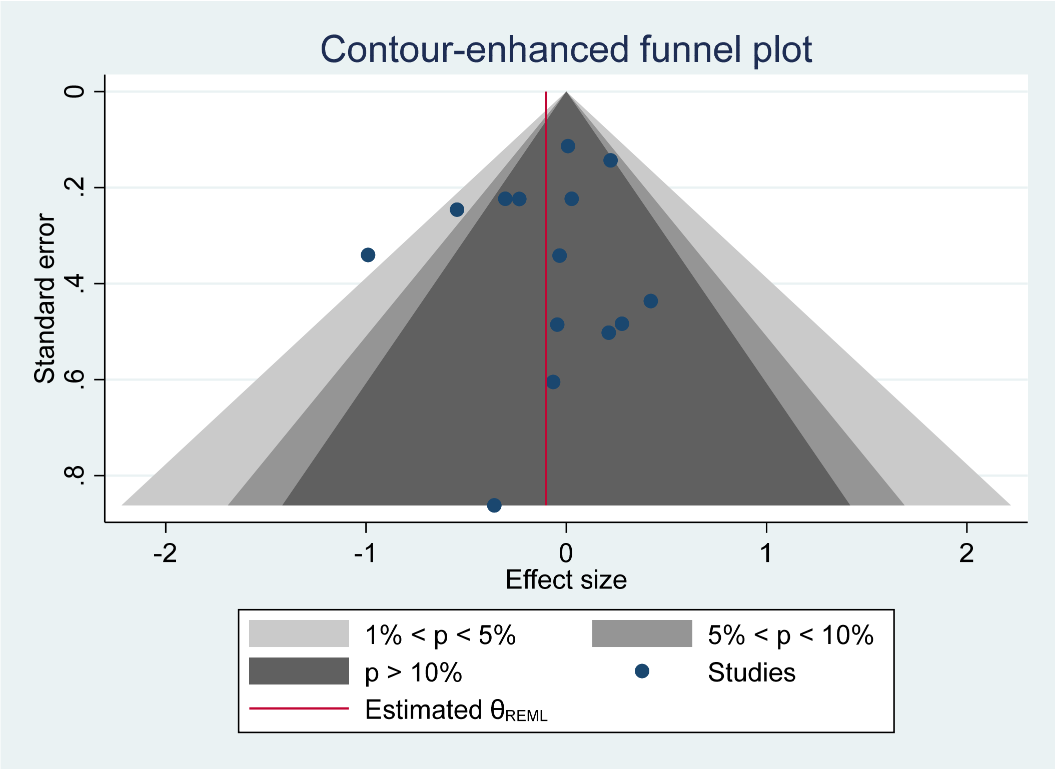

Meta-analysis of linear trend per 50μg/L UIC

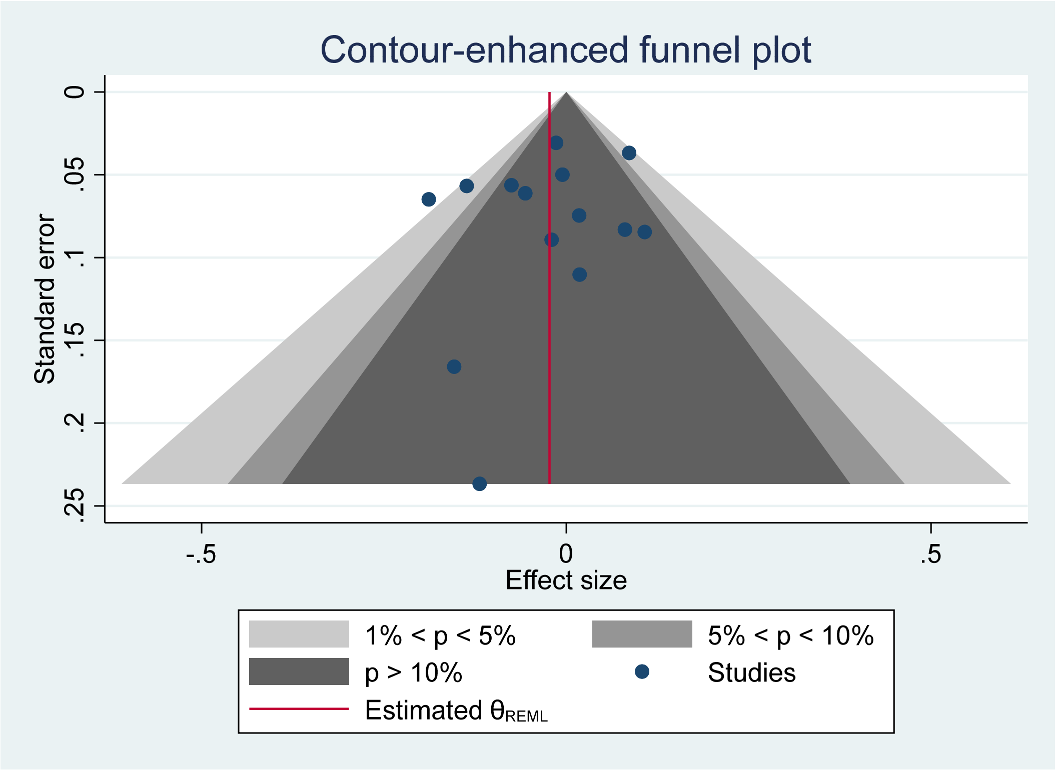

Supplement: Supplementary file 1 [file nutrients-15-00387-s001.zip › nutrients-2120638-supplementary.pdf]
